# The matricellular protein CYR61 promotes breast cancer lung metastasis by facilitating tumor cell extravasation and suppressing anoikis

**DOI:** 10.18632/oncotarget.13677

**Published:** 2016-11-29

**Authors:** Yu-Ting Huang, Qiang Lan, Girieca Lorusso, Nathalie Duffey, Curzio Rüegg

**Affiliations:** ^1^ Department of Medicine, Faculty of Science, University of Fribourg, Fribourg, Switzerland; ^2^ National Center for Competence in Research (NCCR), Molecular Oncology, Swiss Institute for Experimental Cancer Research (ISREC)-Ecole Polytechnique Fédérale de Lausanne (EPFL), Lausanne, Switzerland

**Keywords:** CYR61, metastasis, extravasation, anoikis resistance

## Abstract

Matricellular proteins play multiple roles in primary tumor growth, local invasion and tumor angiogenesis. However, their contribution to metastasis and the putative mechanisms involved are less well characterized. In ER-negative human breast cancer, elevated expression levels of the matricellular protein Cysteine-rich angiogenic inducer 61 (CYR61) are associated with more aggressive progression. Here, we investigated the role of CYR61 in breast cancer lung metastasis using the triple negative human breast cancer cell lines MDA-MB-231 and SUM159. Silencing of CYR61 significantly decreased lung metastasis from tumors orthotopically implanted in pre-irradiated or naive mammary tissue and upon tail vein injection. Constitutive CYR61 silencing impaired cancer cell extravasation to the lung during the first 24 hours after tail vein injection. In contrast, CYR61 inducible silencing starting 24 hours after cancer cell injection had no impact on lung metastasis formation. *In vitro* experiments revealed that CYR61 silencing decreased cancer cell transendothelial migration and motility, reduced CYR61 levels present at the cell surface and sensitized cancer cells to anoikis. Furthermore, we demonstrate that CYR61-dependent cell survival under non-adhesive conditions relied, at least partially, on β_1_ integrin ligation and AMPKα signaling while it was independent of AKT, FAK and ERK1/2 activation. Our data provide the first evidence that CYR61 promotes breast cancer lung metastasis by facilitating tumor cell extravasation and protecting from anoikis during initial seeding to the lung. The uncovered CYR61-β_1_ integrin-AMPKα axis may serve as a potential therapeutic target to prevent breast cancer metastasis to the lung.

## INTRODUCTION

In over 90% of the cases, cancer-related mortality is due to the formation of distant metastasis. Compared with the bulk of cancer cells in the primary site, metastatic cells have stronger abilities to escape the primary tumor, invade distant organs and adapt to novel microenvironmental conditions. Moreover, metastatic cells are often more resistant to anti-cancer therapies, which makes them more difficult to treat [1, 2]. Although our understanding of metastasis formation has significantly improved in recent years, important questions remain unsolved, including in breast cancer [3].

The tumor microenvironment, including extracellular matrix (ECM) proteins, has emerged as a crucial promoter of local tumor progression, invasion and metastasis [4, 5]. Matricellular proteins secreted by cancer cells and stromal cells are non-structural ECM components of the microenvironment. After release into the extracellular space, matricellular proteins bind to cell surface receptors and extracellular matrix proteins to regulate multiple cellular functions, including proliferation, invasion, and metastatic dissemination [6]. Several families of matricellular proteins have been identified, including fibulins, osteopontin, periostin, SPARCs, tenascins, and thrombospondins, and their diversified functions in cancer progression are being gradually unveiled [7].

Cysteine-rich angiogenic inducer 61 (CYR61) belongs to the CCN (CYR61, CTGF and NOV) family of matricellular proteins. It consists of 4 conserved domains: insulin-like growth factor-binding protein (IGFBP), von Willebrand factor type C repeat (VWC), thrombospondin type 1 (TSP-1) repeat and a carboxyl-terminal (CT) domain containing a cysteine knot motif [8]. Several binding sites for integrins and heparin sulfate proteoglycans (HSPG) were identified among the latter three domains [9]. Through binding to different integrins or HSPG, CYR61 can stimulate cell proliferation, survival, adhesion and migration in various types of cells, thereby promoting key cellular events during vascular development, angiogenesis, wound healing and cancer progression [10–14]. The role of CYR61 in cancer progression, however, is highly dependent on the tumor type and the cellular context [10, 15]. Experimental data showed that CYR61 promotes cancer cell growth, migration and invasion in breast, gastric and ovarian cancers, gliomas and pancreatic neuroendocrine tumors [16–24]. Clinical analysis of the correlation between CYR61 levels and tumor stage, recurrence, metastasis and overall survival further confirmed the cancer-promoting role of CYR61 in these types of cancer [20, 21, 25, 26]. In some cancers, however, CYR61 appears to have a tumor-suppressive role. In non-small-cell lung cancer, increased CYR61 expression correlates with reduced tumor growth, invasiveness and progression to late-stages [27, 28]. In endometrial cancer and hepatocellular carcinoma, the role of CYR61 is still under debate since both tumor-suppressive and -promoting effects were proposed [29–32].

In spite of significant correlative and descriptive evidence for a role of CYR61 in promoting metastasis, there is little insight on the putative cellular and molecular mechanisms involved [7]. We have previously reported that tumors growing in a pre-irradiated stroma were selected for tumor cell variants with stronger invasive and metastatic abilities compared to tumors growing in a non-irradiated environment. We found that CYR61 expression was upregulated in more aggressive, selected tumor cell populations and that it contributed to invasion and metastasis through cooperation with α_v_β_5_ integrin [33].

In this study we investigated the role of CYR61 in promoting lung metastasis of triple negative (i.e. ER^−^, PR^−^ and HER2^−^) human breast cancer. We show that CYR61 promotes breast cancer metastasis to the lung following cancer cell implantation in both pre-irradiated and naive mammary fat pad. Through a combination of *in vivo* and *in vitro* experiments we demonstrate that CYR61 facilitates metastasis formation by promoting extravasation of cancer cells into the lung. In addition, we report for the first time that CYR61 suppresses anoikis, through, at least in part, integrin β_1_ and AMPKα-dependent signals.

## RESULTS

### Constitutive and inducible silencing of CYR61 expression in human breast cancer cell lines

To experimentally investigate the role of CYR61 in breast cancer metastasis, we analyzed endogenous levels of CYR61 in different human breast- and breast cancer-derived cell lines: MCF10A, MCF7, MDA-MB-231 and MDA-MB-468. CYR61 expression was low in the non-tumorigenic mammary epithelial cell line MCF10A and in the weakly tumorigenic ER positive MCF7 cell line. In the triple-negative breast cancer cell lines, MDA-MB-468 and MDA-MB-231, CYR61 levels were higher, and highest in the most aggressive and metastatic MDA-MB-231 cells [34] (Supplementary Figure S1A).

We then used shRNA to stably silence CYR61 expression in MDA-MB-231 cells, either constitutively (Supplementary Figure S1B), or in a regulated manner using a doxycycline-inducible shRNA system. For inducible silencing, two different sequences of *CYR61* mRNA targeting shRNAs were combined to obtain the highest silencing efficiency. Non-silencing (NS) shRNA was used as control. Real time PCR analysis of *CYR61* expression in a time course experiment with the inducible system showed that *CYR61* mRNA level in CYR61 knock-down (KD) cells started to decrease one day after addition of doxycycline compared with NS control, and was lowest from day 3 after treatment start (Supplementary Figure S1C). Consistently, the level of CYR61 protein was dramatically decreased 3 days after addition of doxycycline (Supplementary Figure S1D).

To have a second cancer model to consolidate findings in MDA-MB-231 cells, we constitutively silenced CYR61 expression in the metastatic human breast cancer cell line SUM159, originally isolated from a triple-negative breast cancer patient [35]. Compared with the NS control, *CYR61* mRNA targeting shRNAs effectively reduced total CYR61 protein (Supplementary Figure S1E). CYR61 silencing resulted in a reduced cell surface level of CYR61 in both cell lines, as detected by cell surface staining and flow cytometry analysis (Supplementary Figure S1F).

### CYR61 silencing in MDA-MB-231 tumors grown in pre-irradiated mammary fat pads reduces spontaneous lung metastasis formation

**Figure 1:**
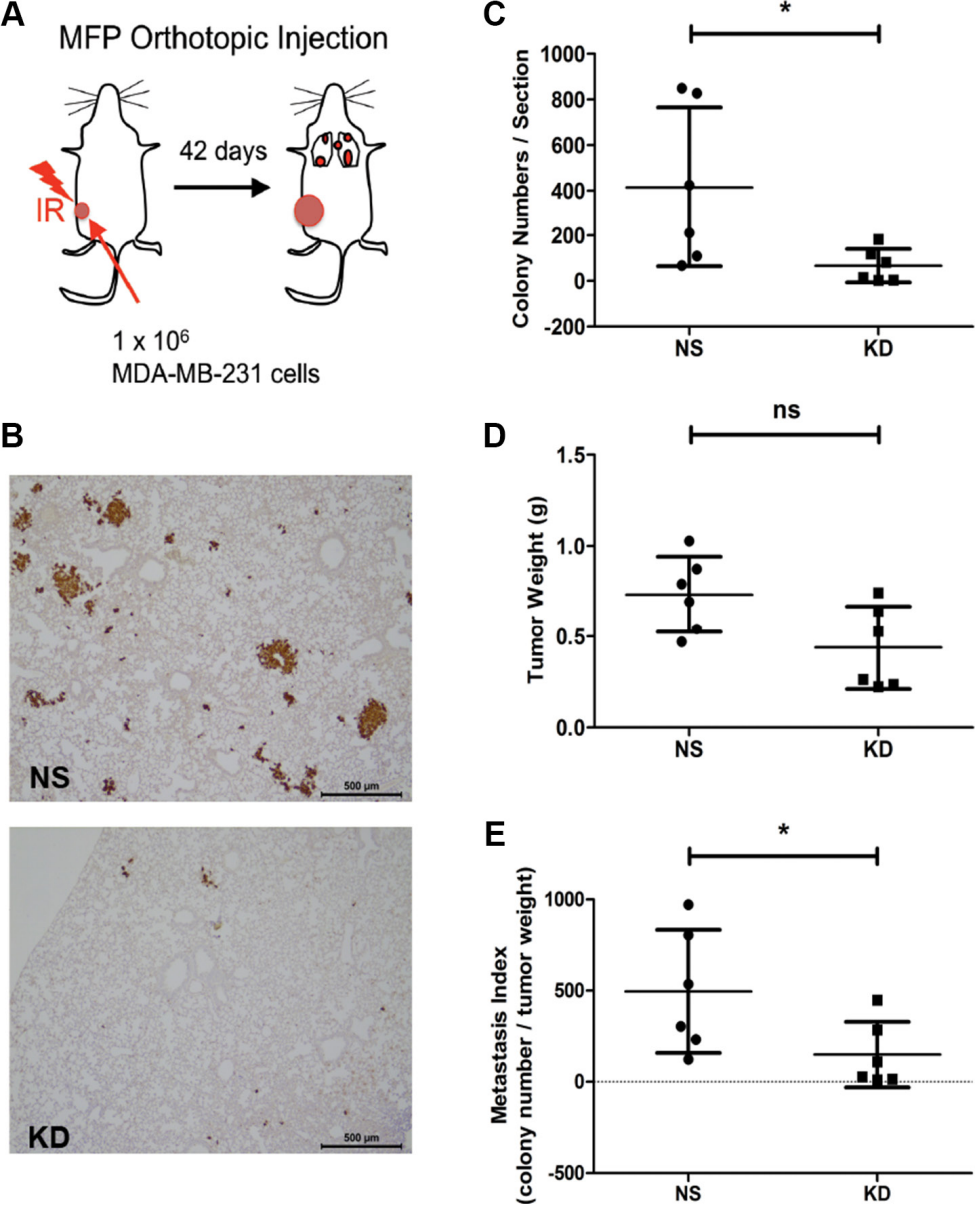
Silencing CYR61 in MDA-MB-231 tumors grown in pre-irradiated mammary fat pads reduces spontaneous lung metastasis formation (**A**) Schematic illustration of the pre-irradiated orthotopic xenograft model of breast cancer. MFP: mammary fat pad. IR: irradiation. (**B**) Immunohistochemical staining of human vimentin to highlight lung metastases. Scale bar: 500 μm. (**C**) Quantification of lung metastasis colony numbers. (**D**) Primary tumor weight in grams (g). (**E**) Metastatic index showing number of metastasis normalized by the tumor weight. *N* = 6 for both groups; *p < 0.05. ns: not significant.

We have previously reported that CYR61 promotes lung metastasis of colorectal and oral squamous cell carcinoma tumors growing subcutaneously in pre-irradiated beds compared to tumors growing in normal, non-irradiated stroma [33]. To test whether CYR61 might also promote metastasis of breast cancer growing in a pre-irradiated bed mimicking the breast cancer tumor microenvironment, we orthotopically injected NS and CYR61 KD MDA-MB-231 cells into 20 Gy pre-irradiated mammary fat pads (MFPs) of NSG mice (Figure 1A). Lung metastases were detected by vimentin staining of lung sections (Figure 1B). Non-silenced tumors growing in pre-irradiated MFP developed more metastatic colonies in the lungs compared to CYR61 silenced tumors (Figure 1C). Many of the lung metastases generated from NS tumors were visibly larger compared to those formed in the KD group. Although CYR61 NS tumors were slightly (but not significantly) bigger than the KD tumors (Figure 1D), the metastatic index (i.e. metastatic colony number normalized by tumor burden) of KD group was still significantly lower than the NS group (Figure 1E). These results demonstrates the role of CYR61 in promoting lung metastasis of breast cancer cells growing in a pre-irradiated mammary bed, a clinically relevant condition observed during post-radiation local relapses, thereby extending previous results obtained in non-orthotopic models [33].

### CYR61 silencing reduces spontaneous lung metastasis formation

**Figure 2:**
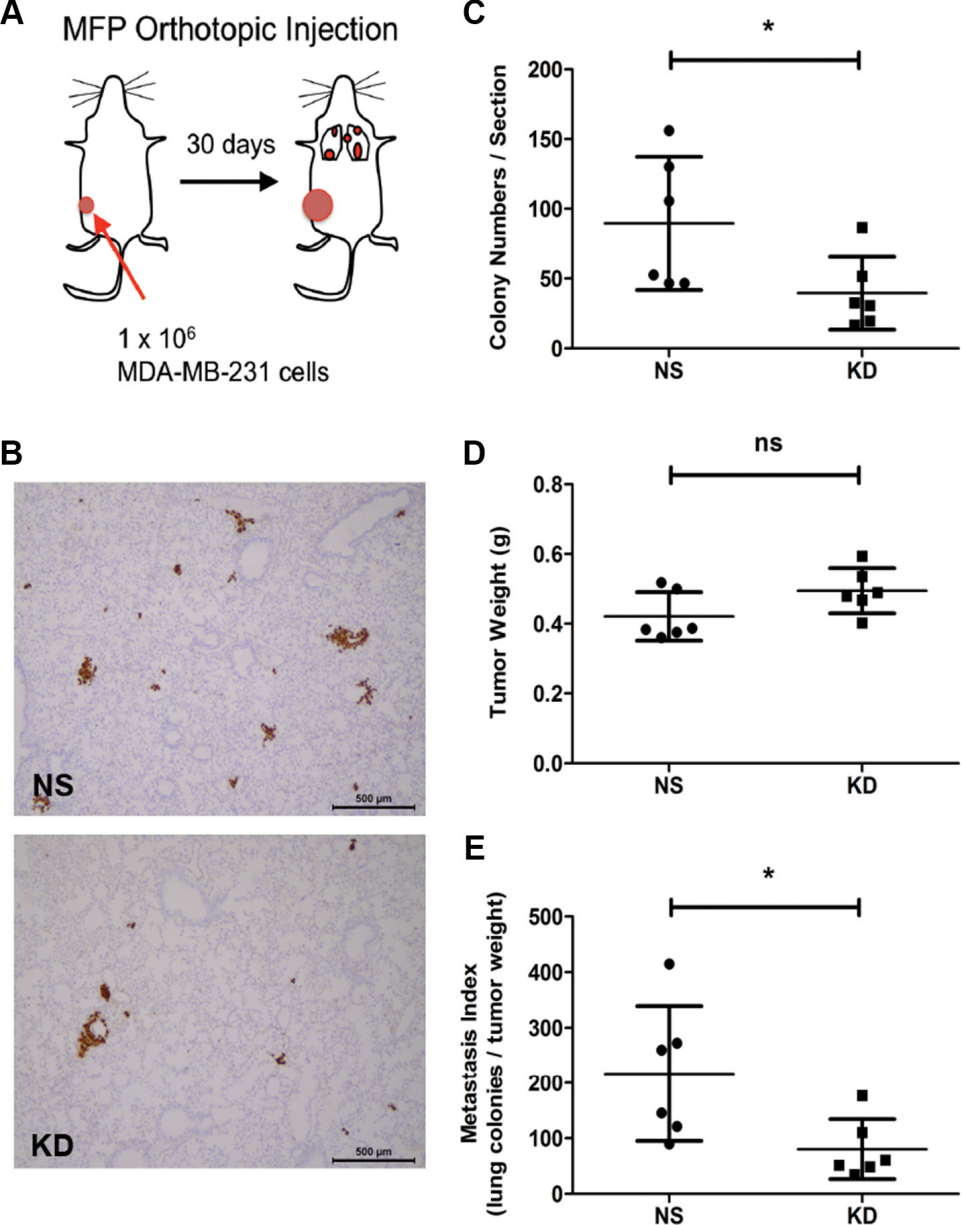
Silencing CYR61 in primary tumors reduces spontaneous lung metastasis formation (**A**) Schematic illustration of the orthotopic injection model. MFP: mammary fat pad. (**B**) Immunohistochemical staining of human vimentin to highlight lung metastases. Scale bar: 500 μm. (**C**) Quantification of lung metastasis colony numbers. (**D**) Primary tumor weight (g). (**E**) Metastatic index showing number of metastasis normalized by the tumor weight. *N* = 6 for both groups; *p < 0.05. ns: not significant.

These results raised the question of whether CYR61 might also promote metastasis formation during the natural course of breast cancer progression. In breast cancer patients, elevated levels of CYR61 expression in the primary tumor correlate with reduced overall survival for all patients, particularly in triple negative cancers [25, 36, 37]. These observations suggested that increased CYR61 expression is associated with metastatic progression in ER negative/basal breast cancers. To experimentally address this question, we orthotopically injected NS and CYR61 KD MDA-MB-231 cells into the MFPs of NSG mice to generate primary tumors and spontaneous metastatic dissemination (Figure 2A). The efficiency of CYR61 knockdown *in vivo* was validated by immunohistochemical staining of CYR61 in primary tumor sections (Supplementary Figure S1G). CYR61 silencing significantly reduced the number of metastatic nodules compared to NS control (Figure 2B and 2C). Primary tumor burden showed no difference between NS and KD groups (Figure 2D). The metastatic index confirmed that CYR61 KD cells had a significantly reduced capacity to spontaneously form lung metastases (Figure 2E).

To confirm the results obtained using MDA-MB-231 cells, we repeated the MFP injection experiment using NS and CYR61 KD SUM159 cells. Although the KD primary tumors grew larger than the NS tumors (Supplementary Figure S2A), we found significantly more metastatic cancer cells in lungs of the NS group (Supplementary Figure S2B and S2C). The metastatic index showed an even more dramatic reduction of the metastatic capacity of CYR61 silenced tumor cells in the SUM159 breast cancer model (Supplementary Figure S3D).

### Down-regulation of CYR61 reduces burden of experimental lung metastasis

**Figure 3:**
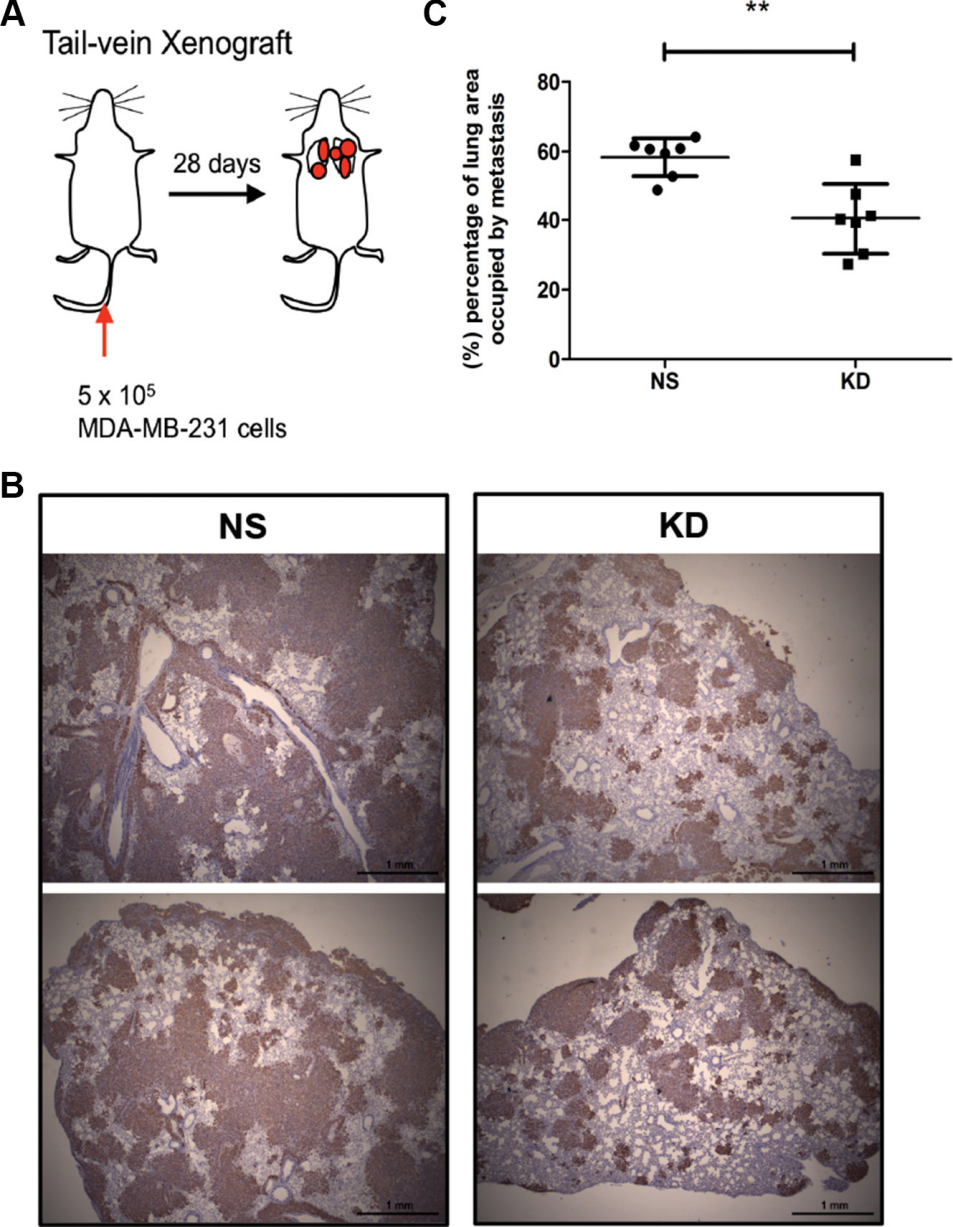
CYR61 down-regulated MDA-MB-231 cells decreases metastatic burden in an experimental model of lung metastasis (**A**) Schematic illustration of the tail vein injection model. (**B**) Immunohistochemical staining of human vimentin to demonstrate lung metastases. (**C**) Quantification of lung area occupied by metastatic tumors. *N* = 7 for both groups; **p < 0.01.

Metastasis develops as a multistep process, also termed the metastatic cascade, during which cells at the primary site have to first locally invade and intravasate, circulate in the blood, then extravasate and colonize the distant organ [38]. We have previously shown that CYR61 promotes tumor cell invasion [24]. To test whether CYR61 also contribute to late steps of metastasis (i.e. extravasation to and colonization of the lung parenchyma), we directly injected NS and KD MDA-MB-231 cells into the tail vein (Figure 3A). Consistent with the orthotopic model, CYR61 silencing significantly reduced lung metastases burden upon intravenous injection (Figure 3B and 3C, KD group vs. NS group). To exclude that reduced lung metastases burden was due to reduced proliferation or increased apoptosis of the cells growing in the lung, we stained colonized lungs for Ki67 and cleaved caspase 3. Results showed no difference between the two groups (Supplementary Figure S3A and S3B). Also no differences in proliferation were seen for both cell lines *in vitro* (Supplementary Figure S3C–S3D).

Collectively, these results indicate that CYR61 promotes breast cancer lung metastasis formation by facilitating the late steps of the metastatic cascade.

### CYR61 facilitates breast cancer cell extravasation into the lung parenchyma

**Figure 4:**
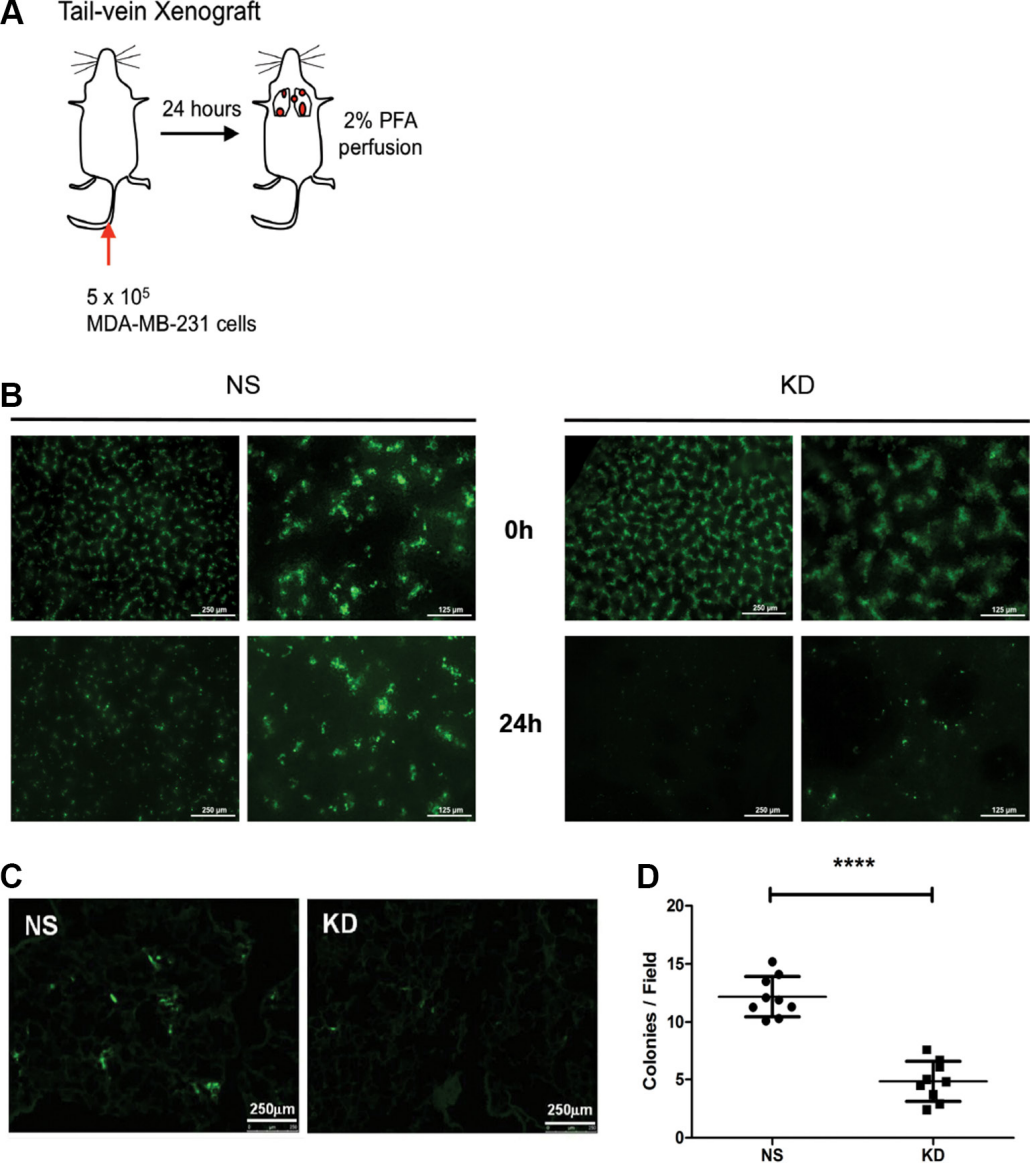
CYR61 facilitates breast cancer cell extravasation into the lung parenchyma (**A**) Schematic illustration of the 24-hour tail vein injection model. At sacrifice, mice were anaesthetized and perfused with 2% PFA to flush out cancer cells still present in the circulation. (**B**) Fluorescent signal from cancer cells labeled with Green Cell Tracker CMFDA at the lung surface. Images taken immediately post-injection (0 hour) show equal amount of NS and CYR61 KD MDA-MB-231 cells. After 24 hours, images demonstrate that significantly more NS cells are present in the lungs compared to KD cells. Scale bar: 125 μm (lower magnification on the left side of panel) and 250 μm (higher magnification on the right side of panel). (**C**) Images from thin sections of lungs obtained 24 hours post-injection confirm the presence of fewer KD cells compared to NS cells. Scale bar: 250 μm. (**D**) Quantification of cancer cell colony numbers per field from experiment in panel (C). *N* = 9 for both groups; ****p < 0.001.

To test whether CYR61 promotes lung metastasis formation by facilitating extravasation into lung parenchyma and/or the successive colonization step, we conducted a short-term tail vein injection experiment. Half-million of NS or KD MDA-MB-231 cells labeled with Green Cell Tracker CMFDA were injected intravenously in NSG mice. A group of mice was anaesthetized, euthanized and immediately analyzed after injection (0 hour, without perfusion). At 24 hours after injection, a second group of mice was anaesthetized, terminally bled and perfused with 2%PFA/PBS to flush cancer cells present in the vasculature before analysis (Figure 4A). From the fluorescent signal at the surface of the intact lung, we found that there were significantly more cancer cells in the lungs of the mice injected with NS MDA-MB-231 compared to mice injected with KD MDA-MB-231 cells (Figure 4B). Immunohistochemical double-staining of CD31 and vimentin on lung sections was performed to visualize endothelial cells and cancer cells. Results show that, at 24 hours after i.v. injection, tumor cells were largely present in the parenchyma outside of the lung vasculature (Supplementary Figure S4A, black arrow). We used frozen sections (Figure 4C) to quantify cancer cells present in the lungs at 24 hours, and, consistent with the surface microscopy, we observed significant fewer extravasated cells in the KD MDA-MB-231 group (Figure 4D). To independently confirm the findings from MDA-MB-231 cells, we performed a similar tail vein injection experiment using NS and CYR61 KD SUM159 cells and obtained consistent results (Supplementary Figure S4B and S4C).

**Figure 5:**
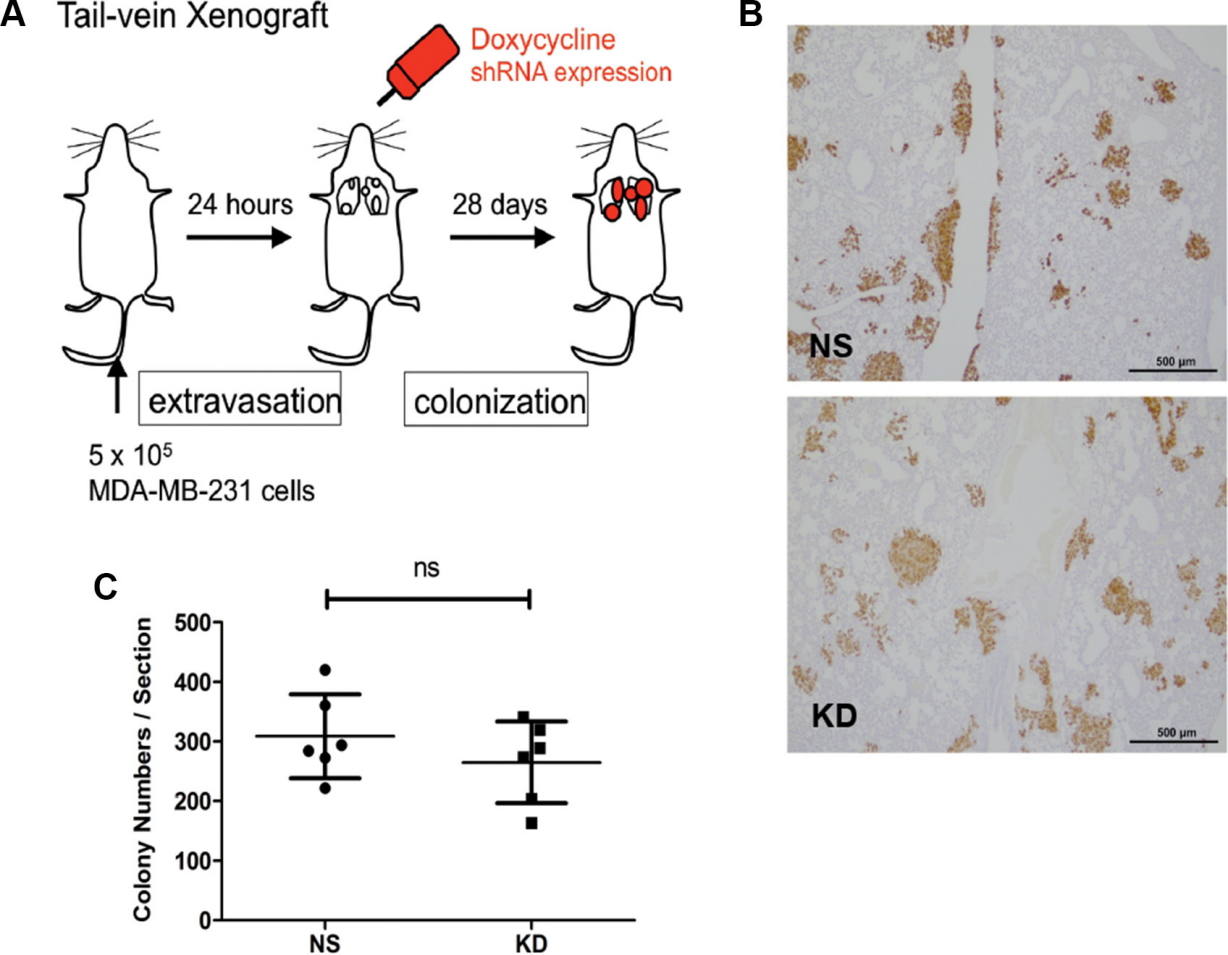
Silencing of CYR61 starting 24 hours after tail vein injection did not affect lung colonization (**A**) Schematic illustration of delayed down-regulation of CYR61 in the tail vein injection model. (**B**) Immunohistochemical staining of human vimentin to highlight lung metastases. Scale bar: 500 μm. (**C**) Quantification of lung metastasis colony numbers reveals no significant difference. *N* = 6 for both groups; ns: not significant.

Next, we used the inducible shRNA knockdown system to further confirm that the effect of CYR61 on facilitating lung metastasis formation was mediated at the early steps of lung seeding. We conducted a tail vein injection experiment whereby CYR61 down-regulation *in vivo* was induced starting from 24 hours after the injection, a time point when most tumor cells have already entered the lung parenchyma (post-extravasation). Doxycycline feeding of the mice was initiated 24 hours after tail vein injection and continued until the mice were sacrificed twenty-four days later to quantify the lung metastasis colonies (Figure 5A). The result of this experiment showed that down regulation of CYR61 starting 24 hours after injection did not impinge on lung metastasis formation (Figure 5B and 5C). CYR61 IHC staining in metastatic lungs confirmed that CYR61 was still efficiently silenced in the lung metastases in the KD group (Supplementary Figure S1H, arrowheads).

Overall, these *in vivo* data indicate that the expression of CYR61 promotes breast cancer cell extravasation into lung parenchyma and thereby affects lung metastasis formation.

### CYR61 facilitates cancer cell migration and transendothelial migration

**Figure 6:**
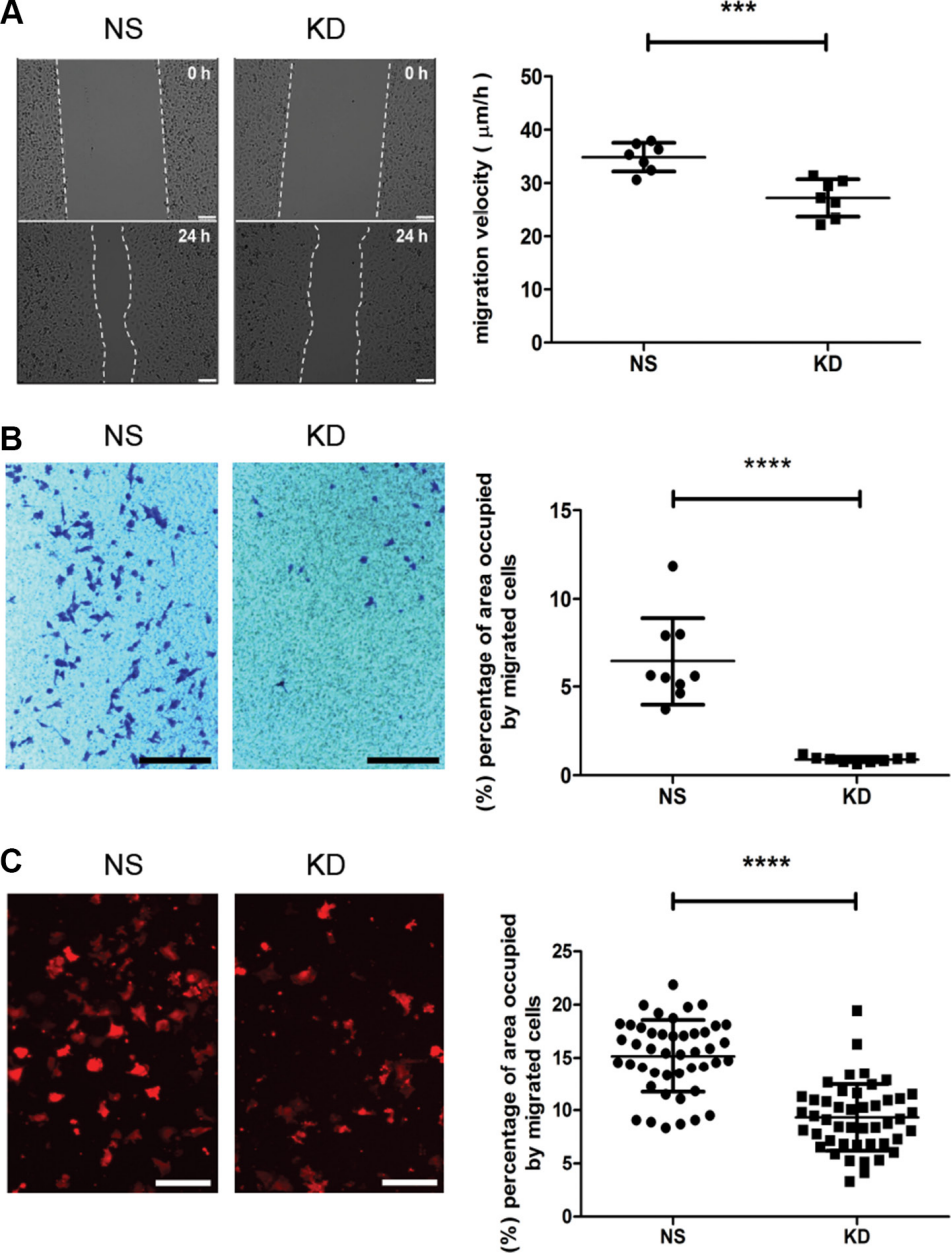
CYR61 facilitates cancer cell migration and transendothelial migration (**A**) CYR61 KD MDA-MB-231 cells migrated slower in a scratch wound assay. Migration velocity was presented as migrated distance per hour (μm/hour) (right panel). Scale bar: 250 μm. (**B**) CYR61 KD MDA-MB-231 cells migrated less efficiently in a transwell migration assay. The surface areas occupied by CV-stained migrated cells are quantified (right panel). Scale bar: 800 μm. (**C**) Reduced transendothelial migration of CYR61 KD MDA-MB-231 cells. The surface areas occupied by migrated cells expressing RFP were quantified (right panel). Scale bar: 125 μm. ***p < 0.005; ****p < 0.001.

To extravasate into secondary organs, circulating cancer cells have to transmigrate through the endothelial layer of the capillary and perivascular space. To further evaluate the contribution of CYR61 to this step, we monitored MDA-MB-231 migration and transendothelial migration *in vitro*. Results from scratch wound and transwell migration assays (Figure 6A and 6B) demonstrated that CYR61 silencing reduced MDA-MB-231 migration. Consistent with these results, pre-incubation of anti-CYR61 antibody significantly inhibited the migration of MDA-MB-231 and SUM159 lines in both assays (Supplementary Figure S5A–S5D). Next, we used a transendothelial migration assay to mimic the process of extravasation occurring *in vivo*, and found that CYR61 KD MDA-MB-231 cells migrated less efficiently through the endothelial monolayer when compared to NS cells (Figure 6C). No differences in proliferation were observed for both cell lines (Supplementary Figure S3C–S3D), thereby excluding the possibility that different proliferation rates might confound the migration results.

Taken together, these results show that CYR61 promotes breast cancer cell migration and transendothelial migration *in vitro*. These results are consistent with the decreased cancer cell extravasation into the lung observed *in vivo*.

### CYR61 protects cancer cells from anoikis

**Figure 7:**
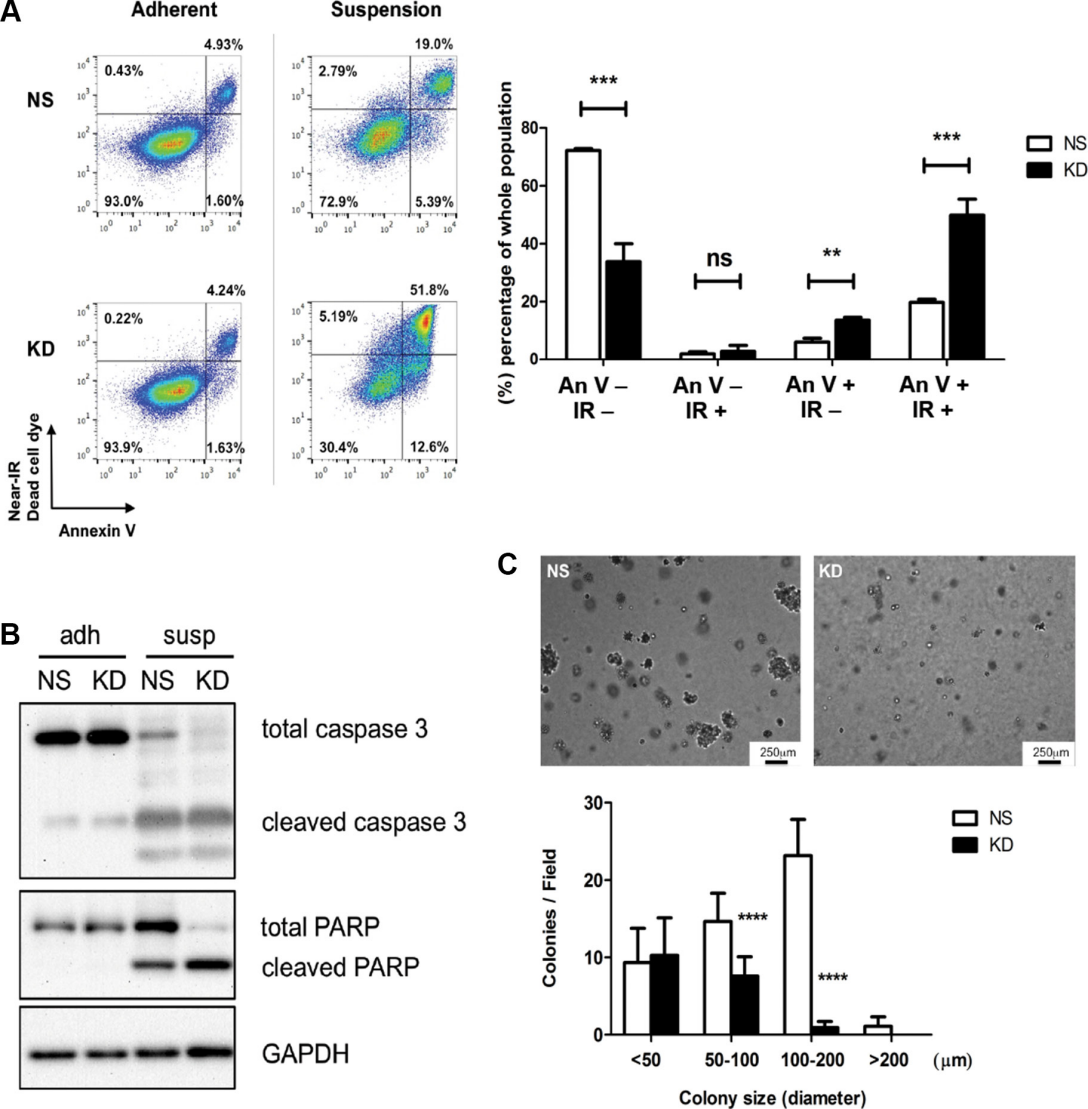
CYR61 protects breast cancer cells from anoikis (**A**) Left panel: NS and CYR61 KD MDA-MB-231 cells under adherent or suspended conditions for 36 hours were stained with Annexin V-Alexa 488 and near-IR dead cell dye and analyzed by flow cytometry. CYR61 KD MDA-MB-231 cells were more sensitive to anoikis and died more compared to NS MDA-MB-231 cells. Right panel: The percentage of viable (Annexin V-/IR-), necrotic (Annexin V-/IR+), early apoptotic (Annexin V+/IR-) and late apoptotic (Annexin-/IR-) cell populations determined by flow cytometry analyses were quantified. (**B**) Western blot analysis showed enhanced ratio of cleaved caspase 3 and PARP to total level in suspended CYR61 KD cells. (**C**) Colony formation assay in agarose demonstrates reduced colony formation of KD MDA-MB-231 cells under anchorage-independent condition. Quantification shows that CYR61 KD MDA-MB-231 cells formed fewer of the larger colonies. Scale bar: 250 μm. **p < 0.01; ***p < 0.005; ****p < 0.001. ns: not significant.

One of the main challenges that cancer cells face while they circulate and before they reestablish adhesive contacts in the secondary organ tissue, is to overcome apoptosis induced by loss of proper cell-ECM adhesion, also known as anoikis [39]. To investigate the effect of CYR61 on cancer cell survival under non-adhesive conditions, we maintained NS and CYR61 KD MDA-MB-231 and NS and CYR61 KD SUM159 cells in suspension and monitored induction of cell apoptosis by Annexin V/Near-IR Dead Cell Marker staining and flow cytometry analysis. The results showed that there were more early and late-apoptotic cells, and fewer viable cells among CYR61 KD cells compared to NS cells (Figure 7A and Supplementary Figure S6A). Induction of anoikis upon cell detachment and its enhancement by CYR61 downregulation was further corroborated by the increased ratio of cleaved versus total forms of caspase 3 and PARP using Western blot analysis (Figure 7B and Supplementary Figure S6B). Moreover, NS MDA-MB-231 cells formed significantly more and larger colonies compared to CYR61 KD cells in the soft agar colony formation assay (Figure 7C), while no difference in growth was observed under conventional 2D-cultural condition (Supplementary Figure S3C).

### CYR61 prevents anoikis, at least partially, through AMPKα and β1 integrin

**Figure 8:**
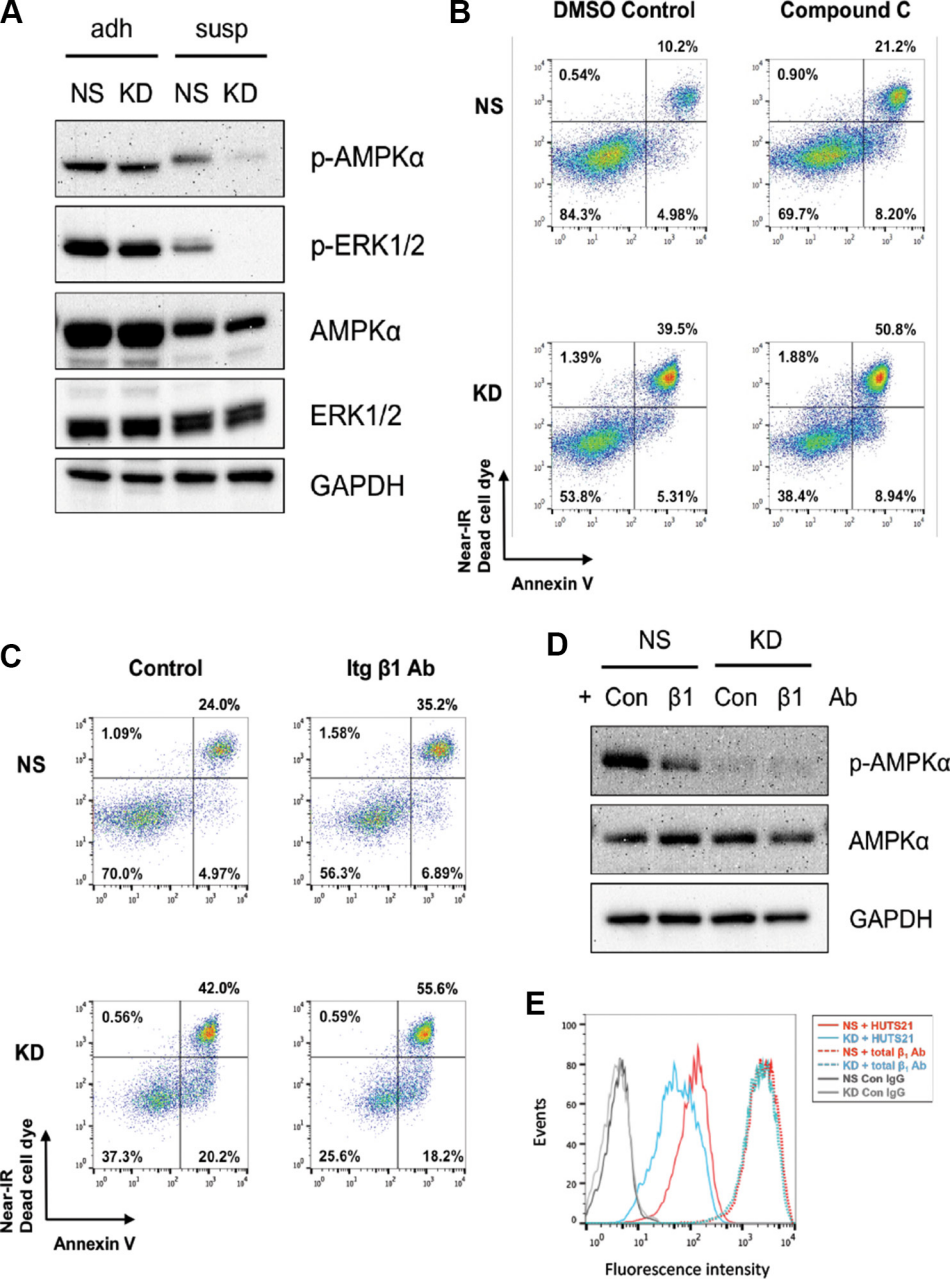
CYR61 suppresses MDA-MB-231 anoikis partially through β_1_ integrin and AMPKα activation (**A**) Western blot analysis showed prolonged phosphorylation of AMPKα and ERK1/2 in suspended NS MDA-MB-231 cells compared to suspended CYR61 KD MDA-MB-231 cells. (**B**) AMPKα inhibition by Compound C (0.1 mM) treatment sensitized cells to anoikis. (**C**) Anti-β_1_ integrin blocking antibody sensitized cells to anoikis. (**D**) Anti-β_1_ integrin blocking antibody reduced the phosphorylation level of AMPKα in NS MDA-MB-231 cells. (**E**) CYR61 silencing reduced the level of activated β_1_ integrin at the surface of KD cells. Cells were stained with antibodies specific for total (dotted lines) or activated (HUTS21, continuous lines) β_1_ integrin. Gray lines stand for the control antibody staining.

Next, we sought to identify the signaling events initiated by CYR61 protecting against anoikis. We analyzed the activation status of various kinases mediating cell survival, in particular AKT, FAK, AMPKα and ERK1/2. Phosphorylation of AKT and FAK were fully abrogated in cells in suspension regardless of CYR61 levels (Supplementary Figure S7A and S7B). In contrast, phosphorylation of AMPKα and ERK1/2 were only partially reduced in suspended NS cells but significantly suppressed in CYR61 KD cells (Figure 8A and Supplementary Figure S8A). Activation of MAPK pathway, especially ERK1/2, has been reported as a mechanism for adherent cells to resist anoikis under non-adhesive conditions [40, 41]. However, when we treated cells in suspension with the MEK1/2 inhibitor U0126 or silenced the ERK1/2-specific phosphatase (DUSP6), neither treatment interfered with cell survival (Supplementary Figure S7C and S7D). In contrast, treatment with the AMPK inhibitor, Compound C, significantly increased the sensitivity to anoikis and enhanced cell death of NS cells in suspension (Figure 8B and Supplementary Figure S8B).

Staining for CYR61 and flow cytometry analysis revealed higher levels of CYR61 on the surface of NS cells (Supplementary Figure S1F). Integrins, including the β_1_ family, are the main cell surface receptors for CYR61 [9]. CYR61 silencing did not impinge on integrin expression (Supplementary Figure S9). However, incubation of cells with an anti-β_1_ blocking antibody increased apoptosis of cells in suspension compared to a control antibody (Figure 8C, Supplementary Figure S8C). Phosphorylation of AMPKα was also reduced in cells treated with anti-β_1_ blocking antibody (Figure 8D and Supplementary Figure S8D). Flow cytometry analysis further showed a higher level of activated form of β_1_ integrin on the surface of NS cells compared to CYR61 KD cells when maintained in suspension (Figure 8E).

Collectively, these data indicate that CYR61 suppresses anoikis, at least partially, through β_1_ integrin and AMPKα activation.

## DISCUSSION

By combining *in vivo* and *in vitro* experiments we demonstrate here that CYR61 promotes lung metastasis of triple-negative breast cancer by facilitating two events relevant to the late steps of metastatic dissemination: firstly, by enhancing extravasation of cancer cells into the lung and, secondly, by conferring resistance to anoikis, at least in part, through the activation of AMPKα pathway but not through AKT, FAK or ERK1/2 signaling.

Most of the published *in vivo* data about CYR61 focus on its function during primary tumor development, especially primary tumor growth, tumor angiogenesis and local invasion. The pro-metastatic ability of CYR61 has been proposed based on enhanced invasiveness using *in vitro* assays [10, 15, 24]. Only rare studies using *in vivo* animal models to investigate the function of CYR61 during metastasis formation have been reported. Among these, one *in vivo* study demonstrated enhanced spontaneous lung metastasis formation by CYR61-overexpressing cells upon orthotopic intratibial injection of osteosarcoma cells [42]. Another report showed that an anti-CYR61 monoclonal antibody blocked lymph node metastasis formation in a footpad tumor inoculation model [43]. By injecting tumor cells through the tail vein, Sun *et al*. demonstrated that overexpression of CYR61 enhanced prostate cancer cell metastasis in bone, lung and the peritoneal cavity while CYR61 down-regulation suppressed it [44]. We previously reported that tumors implanted subcutaneously into a pre-irradiated tumor bed became more invasive and formed more lung metastases compared to tumors growing in a non-irradiated environment. CYR61 was upregulated in these tumors, and was found to contribute to invasion and metastasis through cooperation with α_v_β_5_ integrin [33]. However, the detailed cellular and molecular mechanisms by which CYR61 promoted metastasis were not addressed.

In this paper, we first injected MDA-MB-231 breast cancer cells with high and low levels of CYR61 expression into pre-irradiated MFPs to mimic local breast cancer relapses. Consistent with our previous results from the subcutaneous injection model, MDA-MB-231 cancer cells with high CYR61 expression developed more and larger metastatic colonies in the lung (Figure 1). Importantly, the orthotopic injection of tumor cells in native/intact MFPs revealed a general pro-metastatic effect of CYR61 during the natural course of cancer progression (Figure 2). This is consistent with human data showing that elevated CYR61 expression was associated with poor prognosis [37] and significantly correlated with high expression levels of other tumor-promoting biomarkers [36]. Using the short term experimental metastasis model (i.e. tail vein injection) combined with delayed CYR61 silencing during lung colonization highlighted a critical role of CYR61 in extravasation and the early step of colonization (Figures 3, 4, 5). Data from these systematic *in vivo* experiments collectively provide the first experimental evidence on the role of CYR61 in promoting breast cancer lung metastasis by facilitating cancer cell seeding to the lung. In addition to the *in vivo* validation of CYR61 effects on metastatic seeding, results from *in vitro* assays show that CYR61 enhanced transendothelial migration (Figure 6 and Supplementary Figure S6) as well as resistance to anoikis (Figure 7), which promote two critical capabilities (extravasation and survival during migration) in the early step of metastatic colonization. Although CYR61-enhanced migratory ability has been reported in previous studies, our results show for the first time that CYR61 supports the extravasation step of the metastatic cascade *in vivo*.

Suppression of anoikis is relevant to cancer cell survival during their dissemination in the hematic circulation, when cell-substrate adhesion is absent, transient or weak. This occurs while cells are in the circulation, during transmigration and extravasation to the distant organ and before cells eventually establish firm matrix adhesion once extravasated. To identify signaling events associated with CYR61-dependent resistance to anoikis, we examined the activation status of signaling molecules related to cell survival and used corresponding chemical inhibitors or gene silencing to confirm the causal relation in promoting anoikis resistance. Among the tested pathways, we found sustained higher levels of phosphorylated AMPKα and ERK1/2 in suspended NS cells compared to CYR61 KD cells (Figure 8A and Supplementary Figure S8A). The activation of ERK1/2 has been proposed as a key pro-survival kinase for transformed cells to overcome anoikis due to insufficient glucose uptake and impaired ATP production upon detachment [45, 46]. However, neither blocking ERK1/2 phosphorylation by U0126, or interfering with ERK1/2 dephosphorylation by knocking down DUSP6, a major phosphatase targeting ERK1/2 [47, 48], showed any effect on the sensitivity to anoikis (Supplementary Figure S7C and S7D). Instead, blocking AMPK activation with Compound C significantly increased cell death in suspension (Figure 8B and Supplementary Figure S8B). Together with the blocking experiment with anti-β_1_ integrin antibody and higher activated form of β_1_ integrin at the surface of NS cells (Figure 8C, 8E, and Supplementary Figure S8C), it suggests that CYR61 mediates anoikis resistance through the activation of β_1_ integrin and AMPKα. From the results of sensitizing cells to anoikis by using Compound C and anti- β_1_ integrin blocking antibody, however, the viable populations of treated NS cells are still larger than that from the untreated KD cells, suggesting that additional mechanisms are likely to be involved in protecting cancer cells from anoikis. Nevertheless, β_1_ integrin is implicated in activating AMPKα in suspension as blocking anti-β_1_ integrin decreased AMPKα phosphorylation in NS cells (Figure 8D, 8F and Supplementary Figure S8D). While the activation of AMPKα by CYR61 has been previously reported to enhance cell migration, tube formation and angiogenesis in endothelial cells [49], this is the first report implicating AMPKα activation in CYR61 suppression of anoikis. Based on the fact that CYR61 binds to and signals through integrins, that CYR61-silenced cells have reduced cell surface level of CYR61 and that β_1_ integrin blocking antibody enhances anoikis, we propose that CYR61 prevents anoikis by, at least partially, binding to and activating β_1_ integrin at the cell surface and activating AMPKα in cells that are not adherent. AMPKα is a well-known sensor of the cellular energy status and is activated through binding with AMP and ADP [50]. It was initially recognized as a tumor suppressor due to its function in inhibiting the mTOR pathway and tumor cell proliferation. However, recent findings indicate its contextual oncogenic role by switching between metabolic pathways to keep bioenergetic homeostasis and cell survival under environmental stresses, such as hypoxia, nutrient deprivation and lack of adhesion [51–53]. Indeed, we also found that AMPKα phosphorylation was initially increased in both NS and KD cells once put in suspension. However, by keeping cells in suspension for longer periods of time, NS cells maintained AMPKα phosphorylation at higher levels compared to KD cells (data not shown). Regulation of CYR61 on AMPKα activation could also explain the observation that primary tumors with higher CYR61 levels grew slightly larger, although not statistically significant, in the irradiated MFPs since the microenvironment after irradiation therapy is less perfused and more hypoxic due to suppressed angiogenesis. While under non-irradiated condition, in the absence of hypoxic stress, primary tumor growth showed no apparent differences. These observations are consistent with recent reports showing that cancer cells are able to drive alternative metabolic pathways to escape from traditional treatments and anti-angiogenic therapy [54–56], or to acquire mitochondria DNA from host cells to compensate for mitochondria respiration deficiency [57]. To improve current anti-cancer therapies, both the regulatory mechanisms and the time frame for cancer cells to acquire metabolic switch need to be further elucidated. In a preliminary test by keeping cells under a nutrient-deprived condition, CYR61 expressing NS cells also survived longer when compared with CYR61 KD cells (data not shown). It will be interesting to further investigate whether cells expressing CYR61 could utilize alternative metabolic pathway(s) to survive under stressful microenvironment conditions.

In conclusion, our present results demonstrate that lowering CYR61 level significantly reduces lung metastasis formation of breast cancer by impinging of the late steps of the metastatic cascade. CYR61 promotes cell entering into the lung and suppresses anoikis, the latter, at least in part, though integrin β_1_ and AMPKα-dependent signals. The uncovered CYR61-β_1_ integrin-AMPKα axis may serve as a potential therapeutic target to prevent breast cancer metastasis to the lung.

## MATERIALS AND METHODS

### Cell culture

MCF-7, MDA-MB-468 and MDA-MB-231 cells were grown in DMEM supplemented with 10% FBS and 1% penicillin/streptomycin. SUM159 was maintained in F12 medium with 5% FBS, 5 μg/mL insulin and 1 μg/mL hydrocortisone. MCF-10A was grown in DMEM/F12 medium supplemented with 10% horse serum, 20 ng/mL EGF, 0.5 mg/mL hydrocortisone, 100 ng/mL cholera toxin, 10 μg/mL insulin and 1% penicillin/streptomycin. Human umbilical vein endothelial cells (HUVEC) were grown in M199 medium with 10% FBS, 1% penicillin/streptomycin, 1 μg/mL hydrocortisone, 5 μg/mL EGF, 5 μg/mL bovine pituitary extract, and 25 unit/mL heparin. Cells were passaged every 2–3 days. All cell culture reagents were purchased from Invitrogen Life Technologies (Basel, Switzerland).

### Establishing stable gene-silenced cell lines

The constitutive knockdown shRNA vectors (pLKO.1) and Tet-On inducible knockdown shRNA vectors (pTRIPZ) were purchased from Open Biosystems (Huntsville, AL). Lentiviruses carrying shRNA were produced as described [24] and used to transduce MDA-MB-231 and SUM159 cells. Resistant cells were selected with 4 μg/mL puromycin treatment for 2 days. Pooled cell populations after puromycin selection were used for all the experiments. To induce the expression of shRNA of the pTRIPZ system, 2 or 4 μg/mL doxycycline was added to culture medium of MDA-MB-231 cells and SUM159 cells.

### Real time RT-PCR

Cells were lysed with TriPure Isolation Reagent (Roche, Mannheim, Germany) and total RNA was isolated following manufacturer's instruction. Complementary DNA synthesis and qPCR were done as described [24]. Primer sequences were as following: CYR61 forward: 5′- ACGCTGGATGTTTGAGTGTG. CYR61 reversed: 5′- TGTAGAAGGGAAACGCTGCT. GAPDH forward: 5′- GGACCTGACCTGCCGTCTAG. GAPDH reversed: 5′- CCACCACCCTGTTGCTGTAG.

### Western blot analysis

Cells were lysed with 1% NP-40 RIPA buffer with protease inhibitors. The western blot analysis was done as described [24]. The dilutions of primary and secondary antibodies are 1:1000 and 1:4000. Anti-CYR61 antibody was purchased from Santa Cruz (Heidelberg, Germany, #sc13100). Antibodies against caspase 3, PARP, p-ERK1/2, ERK1/2, p-AMPKα, AMPKα, p-AKT, AKT, p-FAK and FAK were obtained from Cell Signaling (Danvers, MA). GAPDH antibody was purchased from Sigma-Aldrich (Buchs, Switzerland).

### *In vivo* tumor growth and metastasis formation

Eight-week-old NOD-SCID common gamma2-deficient (NSG) female mice (University of Lausanne, Epalinges, Switzerland) were used for all *in vivo* tumor growth and metastasis studies. In pre-irradiation experiments, mice received a single 20 Gy dose delivered to the MFP before cancer cells injection, using an X-RAD iR225 X-ray unit (Precison X-Ray Inc, North Branford, CT) operated at 125 kV, 20 mA, with a 2-mm Al filter, as reported [33]. For orthotopic primary tumor formation, one million luciferase-expressing MDA-MB-231or SUM159 cells were injected into the right 4^th^ mammary fat pad. Formation of lung metastasis was monitored using the Caliper IVIS Lumina II (PerkinElmer, Waltham, MA). For tail vein injection, MDA-MB-231 or SUM159 cells (0.5 million in 100 μL PBS) were injected per mouse. To induce the expression of shRNA of the pTRIPZ system *in vivo*, 2 g/L of doxycycline and 50 g/L of sucrose were added to the drinking water of mice. At the end of the experiments, mice were anaesthetized, terminally bled and perfused with 2% paraformaldehyde (PFA) in PBS. Primary tumors and lungs were fixed in 4% PFA overnight and then embedded in paraffin. For the 24 hours tail vein injection experiments, cancer cells were first labeled with CellTracker Green CMFDA (Thermo Fisher Scientific, Reinach, Switzerland) before injection. At the end of the experiments, lungs were dissected and the fluorescence signal from extravasated cancer cells was measured by Caliper IVIS Lumina II. Lungs were then embedded in OCT for cryosectioning. All animal experiments were done according to national ethical guidelines and were authorized by the veterinary service of Canton Fribourg.

To quantify lung metastases, lung tissue sections were stained by HE or IHC for human vimentin. To determine the total number of metastases, nodules were counted on the whole section under a light microscope (Carl Zeiss, Jena, Germany). To determine the lung area occupied by metastases, five random areas per section were photographed and then quantified using Image J software (https://imagej.nih.gov/ij/). The metastatic index was calculated by normalizing the number of metastatic foci to primary tumor burden.

### Immunohistochemistry (IHC)

The IHC staining was done as previously described [24]. Mouse on mouse blocking reagent for vimentin staining, ABC-HRP kit and ABC-AP kit were purchased from Vector Laboratories (Burlingame, CA). CD31 antibody (Thermo Fisher Scientific, Reinach, Switzerland) and biotinylated goat anti-rabbit secondary antibody (Vector Laboratories) were diluted 1:50 and 1:200, respectively. Vimentin antibody and biotinylated goat anti-mouse secondary antibody (Vector Laboratories) were diluted 1:200 and 1:800, respectively. CYR61 antibody (Santa Cruz Biotechnology, Inc., Heidelberg, Germany, #sc13100) and biotinylated goat anti-rabbit secondary antibody were diluted 1:150 and 1:600, respectively. For CD31/vimentin double-staining, tissues were first stained with anti-CD31 and biotinylated goat anti-rabbit secondary antibody, followed by anti-vimentin-staining and biotinylated goat anti-mouse secondary antibody. Substrates used were DAB (Sigma-Aldrich) and Vector Red (Vector Laboratory).

### Scratch wound migration assay

Scratches on confluent cell monolayer were made with cell culture tips, and the medium was immediately replaced to remove detached cells. The closure of scratch wounds was monitored by time-lapse microscopy for 24 hours using Leica AF6000 (Leica Microsystems, Heerbrugg, Switzerland). Migration velocity was calculated from the migrated distance per hour and presented as μm/h. In CYR61 blocking experiment, cells were pre-incubated with 5 μg/mL anti-CYR61 antibody or control IgG (Sigma, SAB3701275) before conducting the scratches. Representative data from one of the three independent experiments are shown.

### Transwell migration assay

The transwell inserts (8 μm pore size, Corning, Basel, Switzerland) were pre-incubated with serum-free DMEM at 37°C for 30 minutes before use. Then, 700 μL DMEM containing 5% FBS was loaded to the lower part of chamber, and 4 × 10^4^ cells in 200 μL serum-free DMEM was added to the upper part of chamber. Cells were allowed to migrate for 8 hours. Non-migrated cells on the upper side of the filter surface were removed using a cotton swab. Cells migrated to the bottom side of the membrane were fixed with 4% PFA and stained with crystal violet. Three random fields per insert, and in total 9 fields per group, were used to quantify the numbers of migrated cells. In CYR61 blocking experiment, cells were pre-incubated with 5 μg/mL anti-CYR61 antibody or control IgG before loading to the inserts. Representative data from one of the three independent experiments are shown.

### Transendothelial migration assay

HUVECs (5000 cells/insert) were cultured on gelatin pre-coated transwell inserts (8 μm) for 2 days to reach 100% confluence. Medium was replaced with 700 μL M199 containing 5% FBS in the lower chamber, and with 100 μL serum-free M199 in the upper chamber. RFP-expressing cancer cells (4 × 10^4^ cells/100 μL serum-free DMEM) were loaded to each upper chamber and allowed to migrate for 12 hours. Then, HUVECs and non-migrated cancer cells were removed from the upper side of the filter using a cotton swab. Transwell inserts were fixed with 4% PFA, and migrated cells on the lower side of the filter were visualized under a Leica AF6000 fluorescent microscope (Leica Microsystems). Nine random fields from each insert were photographed. In total 27 fields were analyzed by Image J to quantify the area covered by migrated cells. Representative data from one of the three independent experiments are shown.

### Anchorage-independent colony formation assay

Six-well plates (Corning, Basel, Switzerland) were pre-coated with 0.6% agarose in DMEM with 5% FBS. Cells (2 × 10^4^ cells/2 mL/well) mixed with 0.3% agarose in DMEM with 5% FBS were plated. Two mL of DMEM with 5% FBS was added to cover the agarose-cell mixture and the medium were replaced every 2 days. After 2 weeks, wells were photographed (Leica ICC50HD, Leica Microsystems) and number and size of colonies were quantified. Colony size was categorized into 4 groups according to their diameter: < 50 μm, 50–100 μm, 100–200 μm, and > 200 μm. Representative data from one of the three independent experiments are shown.

### Anoikis assay

Cells were trypsinized and plated into poly-HEMA coated plates to induce anoikis. Cells in suspension were stained with Annexin V-Alexa 488 and LIVE/DEAD Fixable Near-IR Dead Cell Stain Kit (Invitrogen Life Technologies, Basel, Switzerland) at the indicated time points. Staining results were detected by FACSCalibur flow cytometer (BD, Mountain View, CA) and analyzed by FlowJo software (Ashland, OR).

### Statistical analysis

Results were presented as mean ± standard deviation (SD). For most experiments, the statistical validation was performed by Mann-Whitney test. Two-way ANOVA was used to analyze the results of Annexin V/IR dead cell dye staining (Figure 6B). *P-value* < 0.05 was considered statistically significant. *p < 0.05 ; **p < 0.01; ***p < 0.001; ****p < 0.0001.

## SUPPLEMENTARY FIGURES


