# Supplementary material for: The matricellular protein CYR61 promotes breast cancer lung metastasis by facilitating tumor cell extravasation and suppressing anoikis
Source: Oncotarget. 2016 Nov 29;8(6):9200–15. doi: 10.18632/oncotarget.13677 (PMC5354725; doi:10.18632/oncotarget.13677)
Supplement: Supplementary file 1 [file oncotarget-08-9200-s001.pdf]

The matricellular protein CYR61 promotes breast cancer lung metastasis by facilitating tumor cell extravasation and suppressing anoikis

Supplementary Materials

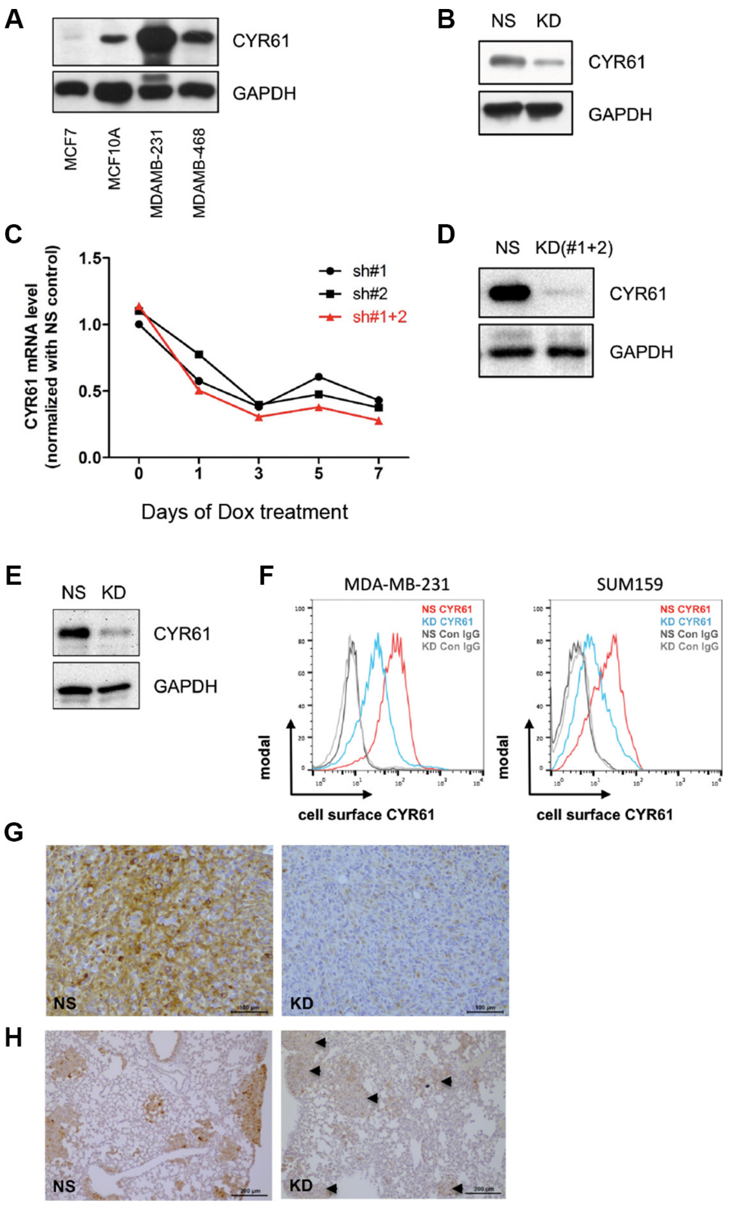

Supplementary Figure S1: Silencing CYR61 expression in MDA-MB-231 and SUM159 cells. (**A**) Representative Western blot showing endogenous CYR61 protein levels in MCF7, MCF10A, MDA-MB-231 and MDA-MB-468 cells. MCF10A is a non-tumorigenic cell line isolated from a fibrocystic patient. MCF7 (ER+), MDA-MB-468 and MDA-MB-231 (triple negative) are tumorigenic. MDA-MB-231 is highly metastatic and also expresses the highest level of CYR61. (**B**) Western blot showing CYR61 silencing efficiency in MDA-MB-231 cells by constitutively expressed shRNA. NS: non-silencing, KD: CYR61 knockdown. (**C**) CYR61 silencing efficiency via inducible CYR61 shRNA by doxycycline (Dox) treatment in MDA-MB-231 cells monitored by CYR61 qPCR. (**D**) Representative Western blot showing silencing efficiency of inducible shRNA in MDA-MB-231 cells. (**E**) Western blot showing silencing efficiency of CYR61 in SUM159 cells by constitutively expressed shRNA. (**F**) Flow cytometry analysis showed reduced surface CYR61 level in CYR61 KD MDA-MB-231 and SUM159 cells. (**G**) Immunohistochemical staining of CYR61 showing the *in vivo* silencing efficiency of the constitutive shRNA in MDA-MB-231 primary tumors grown in mammary fat pads. (**H**) Immunohistochemical staining of CYR61 in MDA-MB-231 lung metastasis showing the *in vivo* silencing efficiency of the doxycycline-inducible shRNA. Arrowheads: Metastatic nodules derived from CYR61 silenced MDA-MB-231 cells.

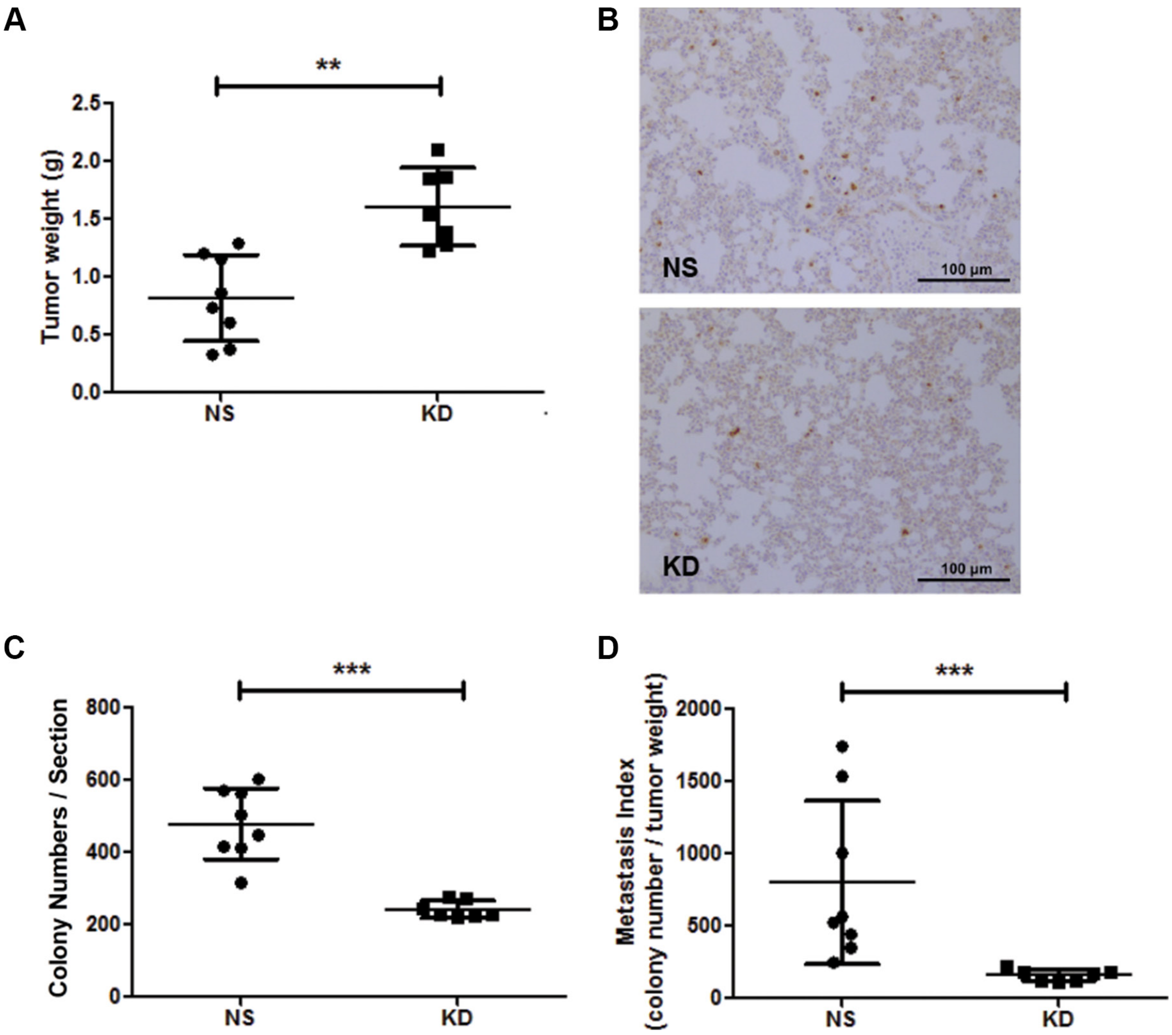

Supplementary Figure S2: Silencing CYR61 in primary tumors reduces spontaneous SUM159 lung metastasis formation. (**A**) Quantification of primary tumor weight. (**B**) Immunohistochemical staining of human vimentin to demonstrate lung metastasis. Scale bar: 100 μm. (**C**) Quantification of lung metastasis colony numbers. (**D**) Metastatic index showing number of metastasis normalized by the tumor weight. $N = 8$ for the NS group, and $N = 7$ for the KD group. $^{**}p < 0.01$; $^{***}p < 0.005$.

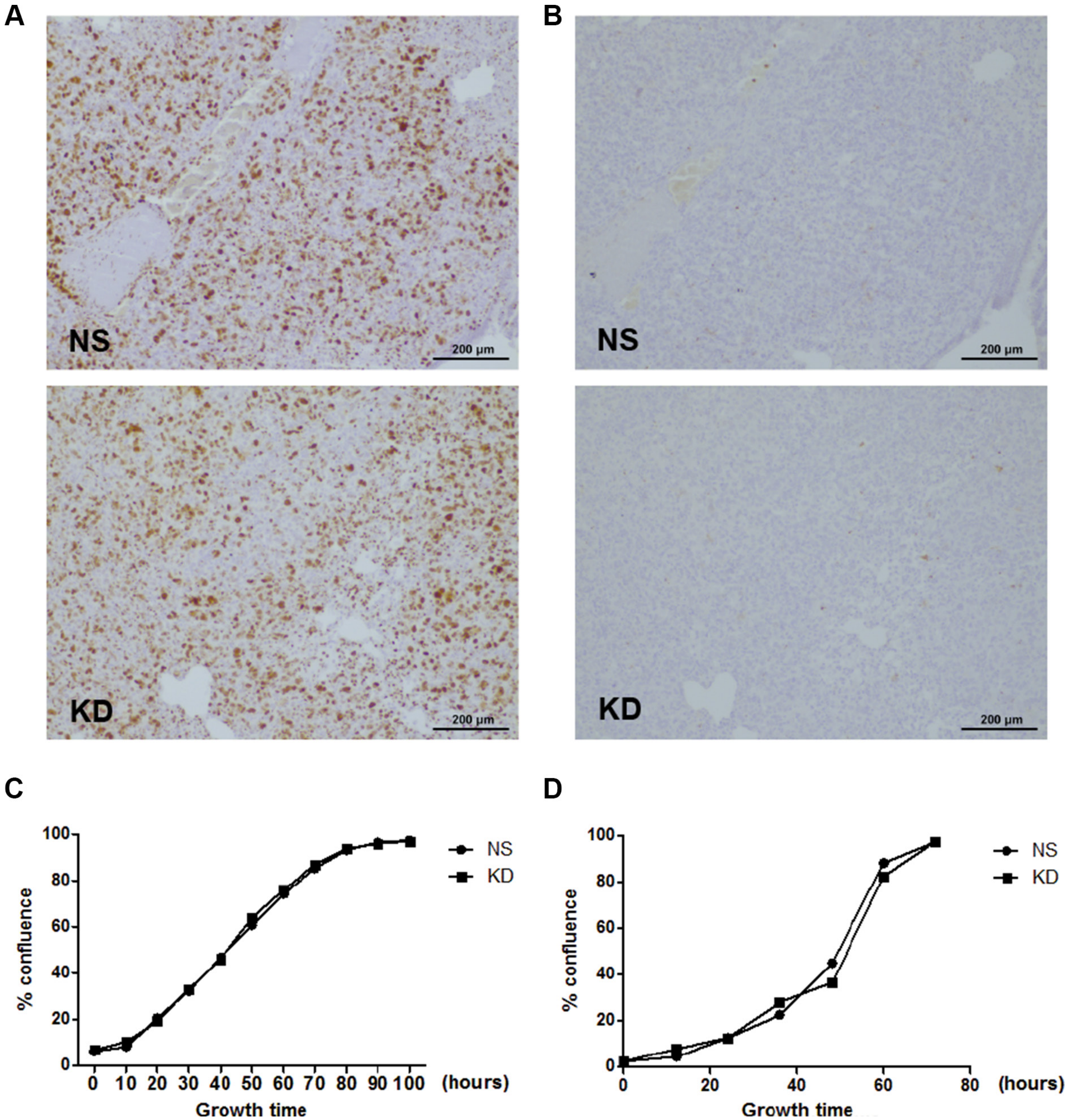

Supplementary Figure S3: Silencing CYR61 showed no difference in cell proliferation or apoptosis *in vivo* and cell proliferation *in vitro*. (**A**) Immunohistochemical staining of Ki67 on lung metastasis sections from Figure 3B (MDA-MB-231) showed no difference in cell proliferation *in vivo* between the NS and KD groups. Scale bar: 200 µm. (**B**) Immunohistochemical staining of cleaved caspase 3 on lung metastasis sections from Fig. 3B showed no difference in cell apoptosis *in vivo* between the NS and KD groups. Scale bar: 200 µm. (**C**) *In vitro* proliferation assay showing no difference between NS and KD MDA-MB-231 cells. (**D**) *In vitro* proliferation assay showing no difference between NS and KD SUM159 cells.

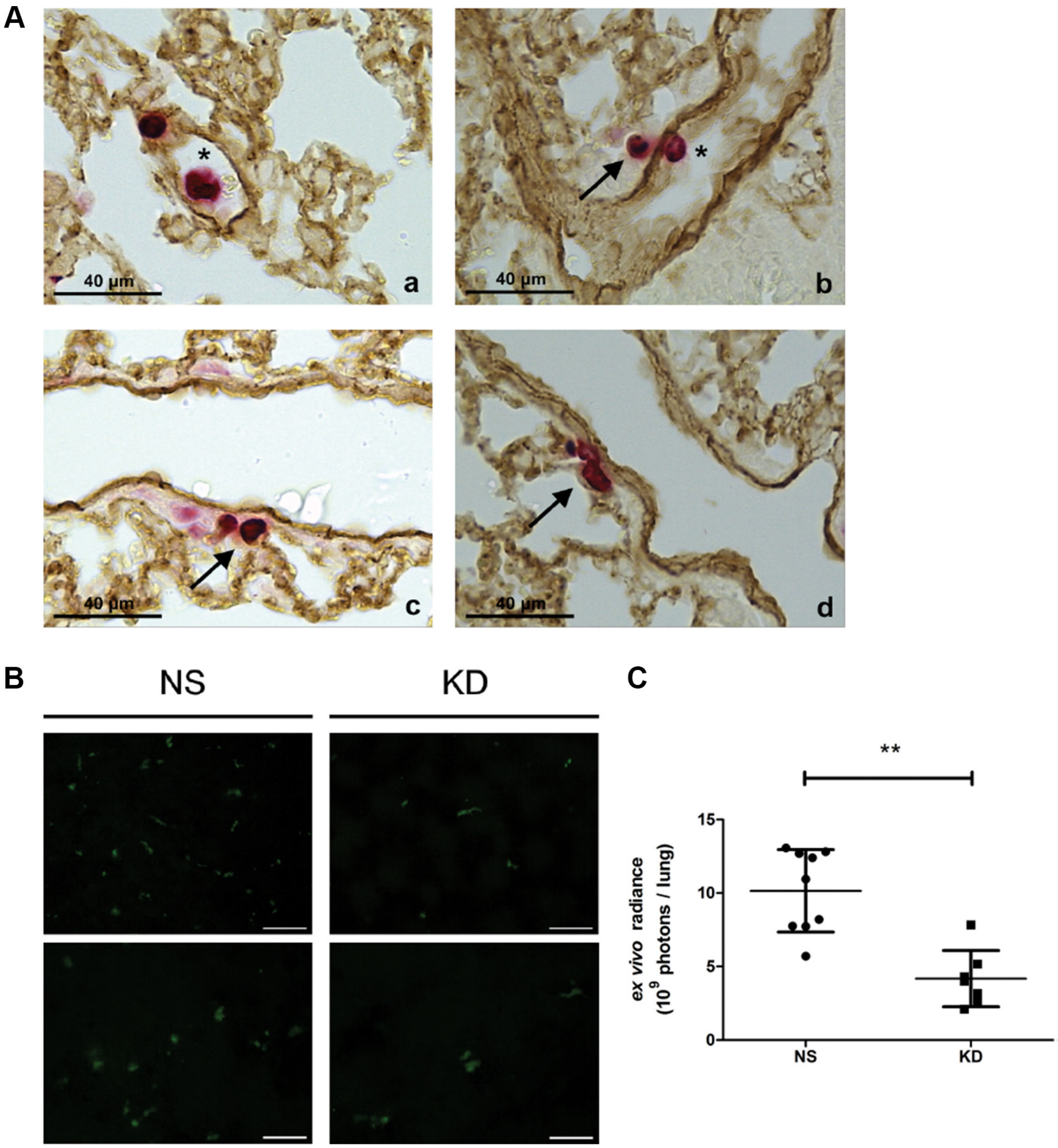

Supplementary Figure S4: Silencing CYR61 reduces cancer cell extravasation to the lung in a 24-hour tail vein injection model. (**A**) Lung sections were double-stained with CD31 (brown) and vimentin (red) for endothelial and NS MDA-MB-231 cells, respectively. (a–b) Most cells were stained inside the vessels from the lung sections of mice sacrificed 30 minutes after injection without perfusion (asterisks). (c–d) Cancer cells were mostly located outside of vessels in perfused lungs 24 hours after injection (black arrows). (**B**) NS and CYR61 KD SUM159 cells labeled with Green Cell Tracker CMFDA were injected into the tail vein of NSG mice. After 24 hours, mice were anesthetized and perfused with 2% PFA/PBS to remove cells from the circulation. Images of the lung surface demonstrate that significantly more NS cells have extravasated into the lungs. Scale bars: 200 µm (upper panels); 100 µm (lower panels). (**C**) *Ex vivo* measurement of fluorescent signal from extravasated cells in lungs from (B). $N = 9$ for NS group and $N = 7$ for KD group; $^{**}p < 0.01$.

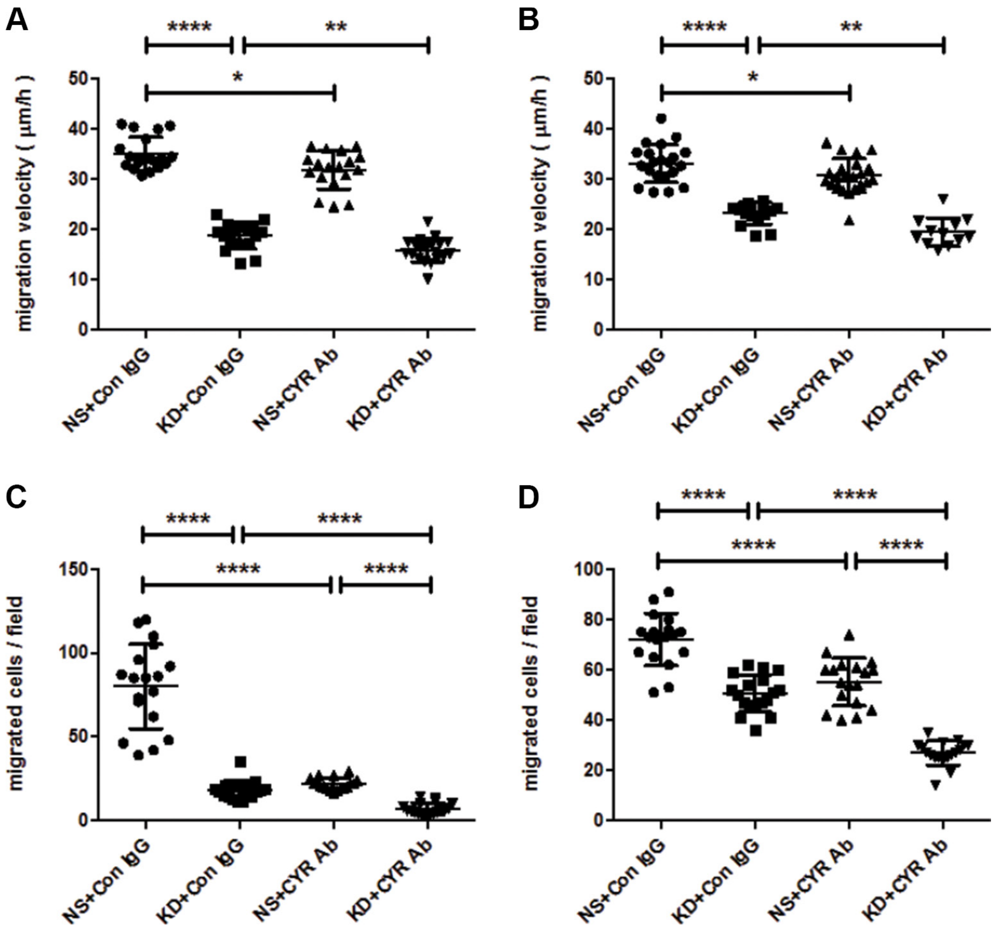

Supplementary Figure S5: Cell migration is decreased by anti-CYR61 antibody. (**A**) MDA-MB-231 and (**B**) SUM159 cell lines pre-treated with anti-CYR61 antibody, migrated slower in a scratch wound assay. Migration velocity was presented as migrated distance per hour (μm/hour). (**C**) MDA-MB-231 and (**D**) SUM159 cell lines pre-treated with anti-CYR61 antibody, migrated slower in a transwell migration assay. Control cells were treated with a non-specific IgG. For quantification, the numbers of migrated cells in each randomly chosen field were counted. $*p < 0.05$; $***p < 0.005$; $****p < 0.001$.

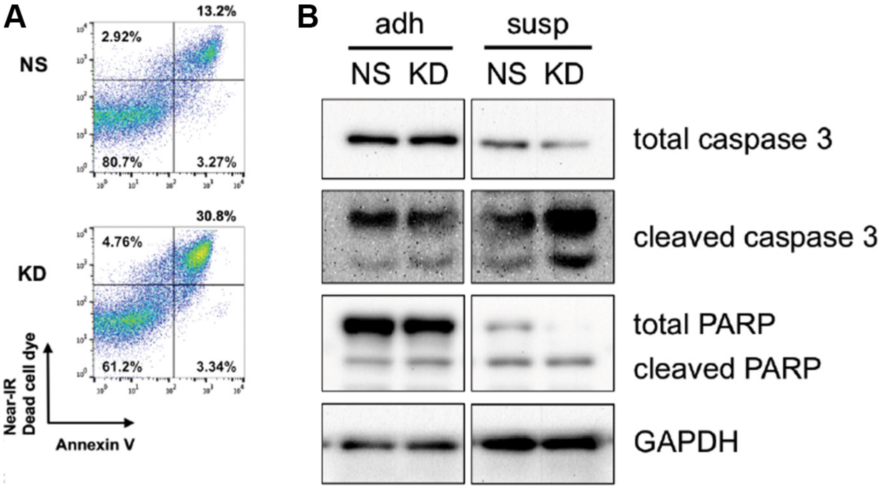

Supplementary Figure S6: CYR61 protects SUM159 cells from anoikis. (**A**) NS and CYR61 KD SUM159 cells under suspension for 24 hours were stained with Annexin V-Alexa 488 and near-IR dead cell dye and analyzed by flow cytometry. CYR61 KD cells were more sensitive to anoikis and died more efficiently. (**B**) Western blot analysis showing enhanced ratio of cleaved caspase 3 and PARP to total levels in suspended CYR61 KD SUM159 cells.

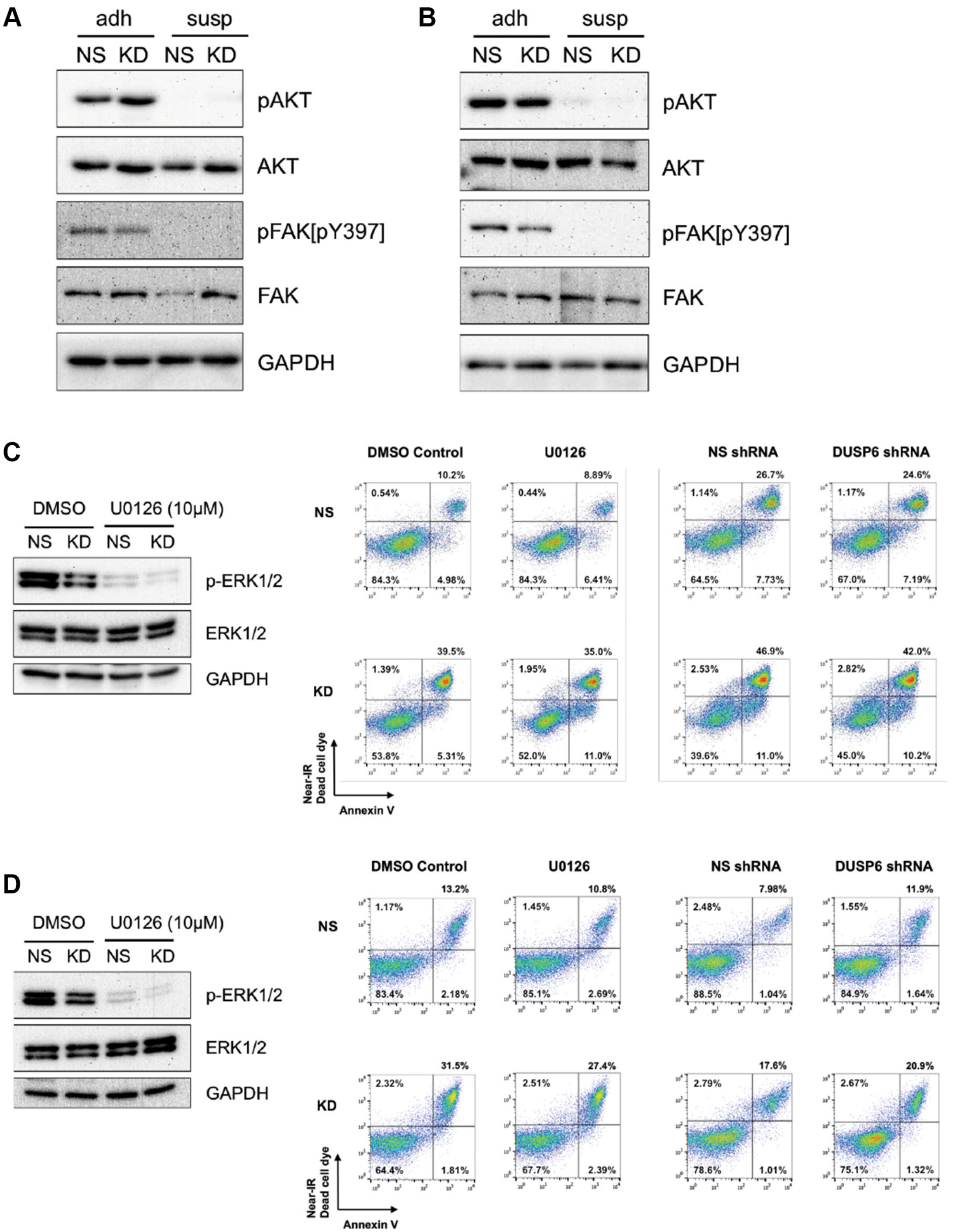

Supplementary Figure S7: CYR61-dependent resistance to anoikis is independent of AKT, FAK and ERK1/2 activation. (**A**–**B**) Western blot analysis showing that phosphorylation of AKT and FAK were completely lost in (A) MDA-MB-231 cells and (B) SUM159 cells under suspension regardless of CYR61 expression levels. (**C**–**D**) Treatment of U0126 (10 μM), the chemical inhibitor of MEK1/2, or silencing DUSP6, the dominant phosphatase of ERK1/2, did not affect anoikis resistance in (C) MDA-MB-231 cells or (D) SUM159 cells.

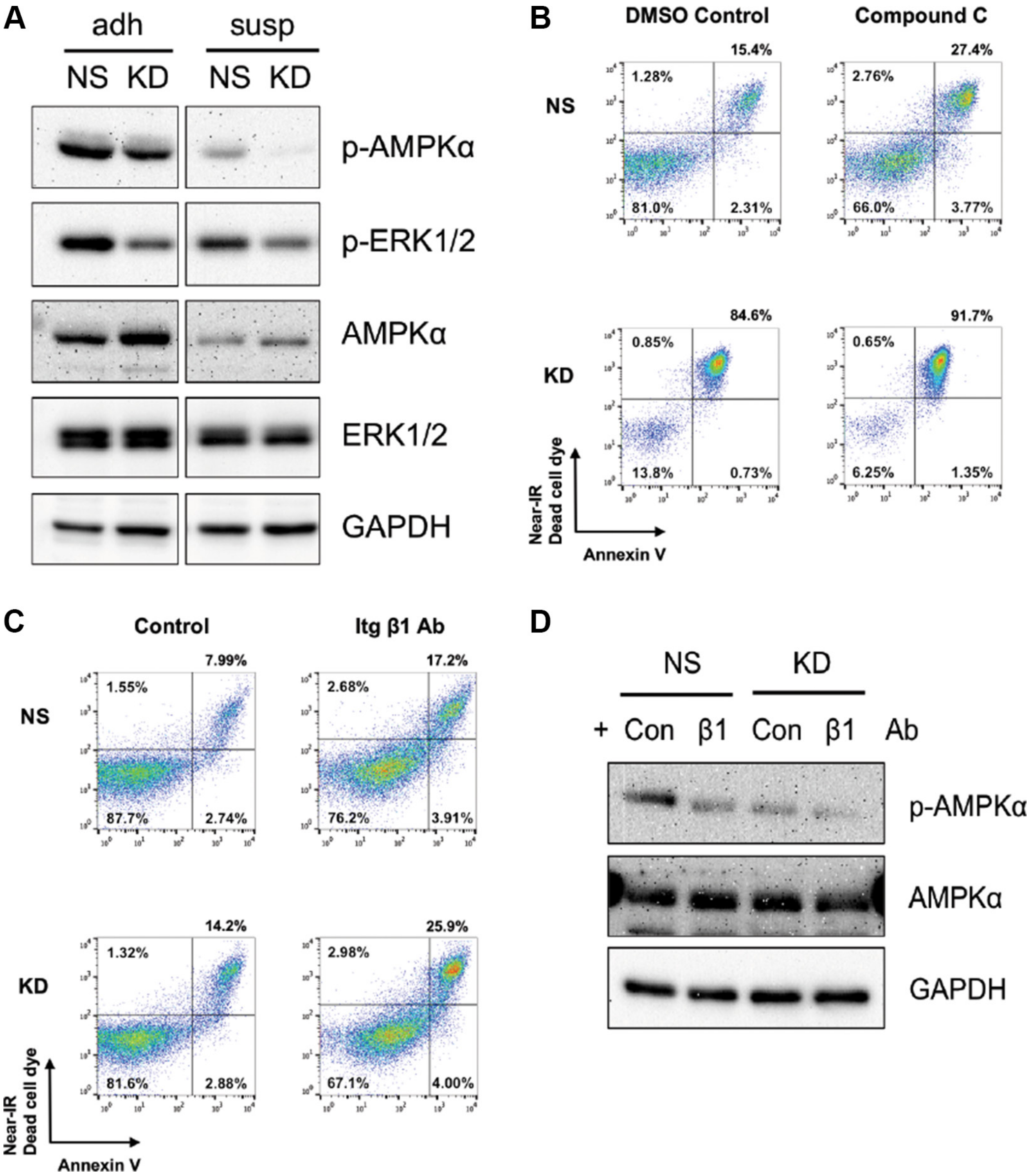

Supplementary Figure S8: CYR61 suppresses SUM159 cell anoikis partially through β1 integrin and AMPKα activation. (**A**) Western blot analysis showing prolonged phosphorylation of AMPKα and ERK1/2 in suspended NS SUM159 cells compared to suspended CYR61 KD SUM159 cells. (**B**) Blocking AMPKα activation by Compound C (0.1 mM) treatment increased SUM159 cell sensitivity to anoikis. (**C**) Anti-β_1 integrin blocking antibody sensitized SUM159 cells to anoikis. (**D**) Anti-β_1 integrin blocking antibody reduced the phosphorylation level of AMPKα in SUM159 cells.

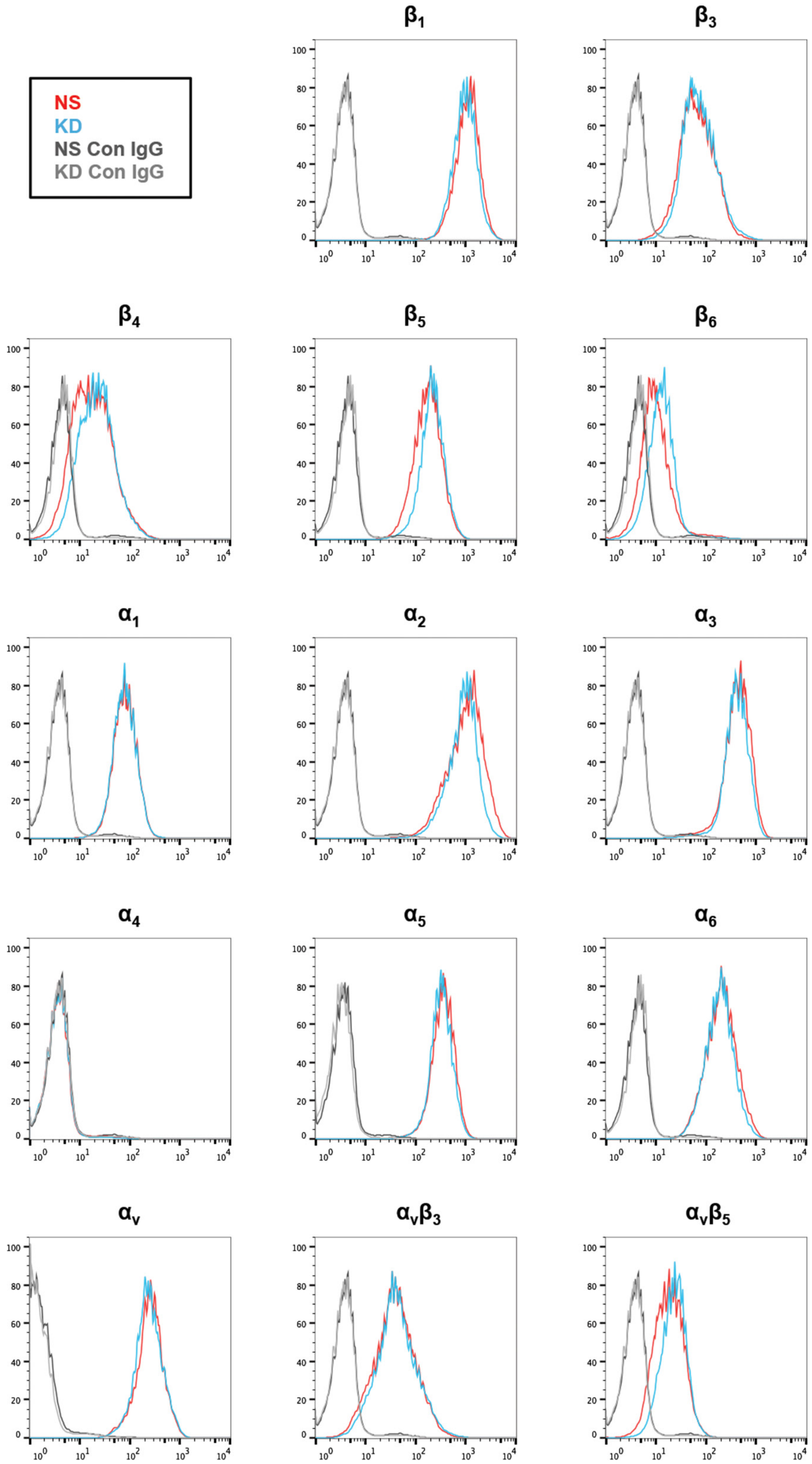

Supplementary Figure S9: The expression profile of integrin subunits in NS and CYR61 KD MDA-MB-231 cells. The NS and CYR61 KD MDA-MB-231 cells were stained either with specific anti-integrin subunit antibodies or with control IgG antibodies as indicated and then analyzed by flow cytometry. CYR61 silencing has no effect on cell surface integrin expression.
